# Inhibition of cell surface GRP78 and activated **α**2M interaction attenuates kidney fibrosis

**DOI:** 10.1172/jci.insight.183998

**Published:** 2025-12-22

**Authors:** Jackie Trink, Ifeanyi Kennedy Nmecha, Katrine Pilely, Renzhong Li, Zi Yang, Sydney Kwiecien, Melissa MacDonald, Bo Gao, Mariam A. Mamai, Chao Lu, Urooj F. Bajwa, Nikhil Uppal, James C. Fredenburgh, Masao Kakoki, Salvatore V. Pizzo, Anthony F. Rullo, Matthew B. Lanktree, Jeffrey I. Weitz, Yaseelan Palarasah, Joan C. Krepinsky

**Affiliations:** 1 Division of Nephrology, Department of Medicine, McMaster University, Hamilton, Ontario, Canada.; 2 Department of Molecular Medicine-Cancer and Inflammation, University of Southern Denmark, Odense, Denmark.; 3 Department of Chemistry and Chemical Biology and; 4 Department of Health Research Methods, McMaster University, Hamilton, Ontario, Canada.; 5 Thrombosis and Atherosclerosis Research Institute (TaARI), Hamilton, Ontario, Canada.; 6 Department of Physiology, Dokkyo Medical University, Mibu, Japan.; 7 Department of Pathology, Duke University Medical Center, Durham, North Carolina, USA.

**Keywords:** Nephrology, Therapeutics, Chronic kidney disease, Drug therapy, Fibrosis

## Abstract

We recently showed that cell surface translocation of the endoplasmic reticulum–resident protein GRP78, when bound by activated α 2-macroglobulin (α2M*), induces pro-fibrotic responses in glomerular mesangial cells in response to high glucose and regulates activation of the pro-fibrotic cytokine transforming growth factor-β1 (TGF-β1), implicating a pathogenic role in glomerulosclerosis. Interstitial fibrosis, largely mediated by proximal tubular epithelial cells (PTEC) and renal fibroblasts, develops later in kidney disease and correlates with functional decline. Here we investigated whether interstitial fibrosis was mediated by cell surface GRP78 (csGRP78)/α2M*. High glucose and TGF-β1 increased csGRP78 and α2M* in PTEC and renal fibroblasts, and their inhibition prevented fibrotic protein production. Interestingly, for TGF-β1, this depended on inhibition of noncanonical signaling through YAP/TAZ, with Smad3 activation unaffected. In vivo, type 1 diabetic Akita mice overexpressing TGF-β1 were treated with either a neutralizing antibody for csGRP78 (C38) or α2M* (Fα2M) or an inhibitory peptide blocking csGRP78/α2M* interaction, and mice with unilateral ureteral obstruction were treated with Fα2M or inhibitory peptide. Consistently, inhibition by antibody or peptide attenuated fibrosis and pro-fibrotic signaling. These findings show an important role for csGRP78/α2M* in mediating tubulointerstitial fibrosis in both diabetic and nondiabetic kidney disease and support their inhibition as a potential antifibrotic therapeutic intervention.

## Abstract 2

Blocking the interaction between two proteins that are increased in diabetic and nondiabetic kidney disease (cell surface GRP78 and alpha 2-macroglobulin) inhibits kidney fibrosis.

## Introduction

Chronic kidney disease (CKD) affects more than 10% of the world’s population and is associated with a significant increase in morbidity and mortality (1). It is characterized by the development of glomerular sclerosis and tubulointerstitial fibrosis, which eventually leads to loss of kidney function and end-stage kidney disease in many individuals. This requires dialysis or transplantation to sustain life but is marked by significantly reduced quality of life and a substantial economic burden on the health care system. The most common causes of CKD in North America are diabetes and hypertension, with control of glucose and blood pressure as important factors constituting the current standard of care (2). This also includes inhibitors of the renin-angiotensin system and the sodium glucose co-transporter 2. However, current therapies are unable to prevent disease progression. There is thus a critical need for novel therapeutics with a unique mechanism of action.

Our lab has previously shown that high glucose induces the translocation of GRP78, an endogenous endoplasmic reticulum protein, to the cell surface of glomerular mesangial cells (3). Here, GRP78 acts as a pro-fibrotic signaling receptor. In parallel, we showed that the known ligand for cell surface (cs) GRP78, activated α 2-macroglobulin (α2M*), is locally produced by mesangial cells in response to high glucose. Its interaction with csGRP78 promotes downstream pro-fibrotic signaling, key to the development of early diabetic kidney disease and to the development and progression of glomerulosclerosis in nondiabetic CKD (3, 4). We further showed that this signaling pathway can mediate synthesis and activity of the pro-fibrotic cytokine transforming growth factor β1 (TGF-β1). Although it is well known to be a major contributor to CKD of varying etiology (5, 6), the pleiotropic homeostatic functions of TGF-β1 make its direct inhibition infeasible (7). The indirect inhibition of TGF-β1 activity is thus of current therapeutic interest, with the targeting of csGRP78/α2M* signaling a potential novel approach. Importantly, we have shown that csGRP78 and α2M synthesis and activation are only present in a diseased state, making their inhibition an attractive therapeutic target with potentially limited side effects.

The progression of both diabetic and nondiabetic CKD is marked by the development of tubulointerstitial fibrosis, a process largely regulated by crosstalk between proximal tubule epithelial cells (PTEC) and renal fibroblasts (8–10). The relevance of csGRP78/α2M* signaling to the regulation of tubulointerstitial fibrosis is as yet unknown. Here, we wished to determine whether this ligand/receptor pair could also mediate tubulointerstitial fibrosis, thereby supporting its targeting as a more generalizable approach to reducing fibrosis in CKD. Further, given the prominent role of TGF-β1 in the development of kidney fibrosis, we also wished to determine whether csGRP78/α2M* could mediate the cellular pro-fibrotic response to direct TGF-β1 treatment in the absence of high glucose. Importantly, the therapeutic potential of inhibiting csGRP78/α2M* signaling was explored in vivo in both diabetic and nondiabetic models of kidney fibrosis.

## Results

### High glucose–induced csGRP78 and α2M* mediate matrix production in PTEC and renal fibroblasts.

**Figure 1:**
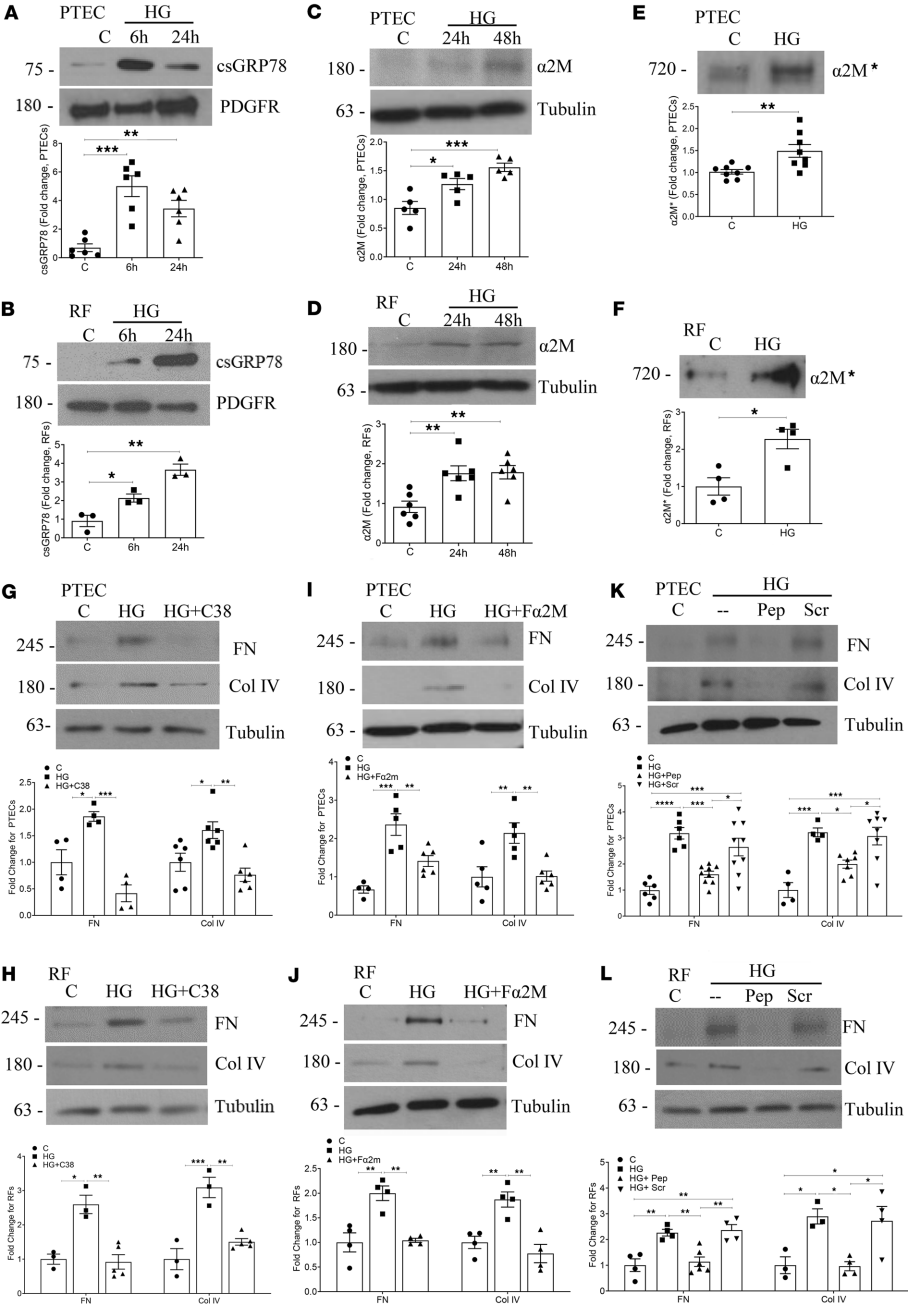
Inhibition of csGRP78 or α2M* prevents high glucose–induced matrix production in PTEC and renal fibroblasts. High glucose– (6 or 24 hours, 30 mM) induced csGRP78 expression, assessed by biotinylation, was increased in (**A**) PTEC (*n* = 6) and (**B**) renal fibroblasts (*n* = 3). Production of α2M (24 and 48 hours) by PTEC (**C**) and renal fibroblasts (**D**) was increased by high glucose (30 mM, *n* = 5 and 8, respectively). Similar results were observed for α2M activation (**E** and **F**, respectively) (high glucose 48 hours, 30 mM, *n* = 5 and 4, respectively). Inhibition of csGRP78 interaction with α2M* using the GRP78-targeting antibody C38 prevented high glucose– (30 mM, 48 hours) induced fibronectin and collagen IV production in both (**G**) PTEC (*n* = 4–6) and (**H**) renal fibroblasts (*n* = 3–5). Similarly, α2M* inhibition with the Fα2M antibody attenuated matrix protein production in high glucose (30 mM, 48 hours, *n* = 5–6 PTEC and 4 renal fibroblasts (**I** = PTEC and **J** = renal fibroblasts). Peptide inhibition of the csGRP78/α2M* interaction also prevented matrix protein production in high glucose (30 mM, 48 hours) by (**K**) PTEC (*n* = 4–9) and (**L**) renal fibroblasts (*n* = 3–6) (*P < 0.05, **P < 0.01, ***P < 0.005, ****P < 0.001).

We previously showed the importance of high glucose–induced pro-fibrotic csGRP78/α2M* signaling in mesangial cells (4–6). While this is relevant to the development of glomerulosclerosis in diabetic kidney disease, whether this ligand-receptor pair also regulates tubulointerstitial fibrosis is not known. We thus first assessed whether csGRP78 and α2M* are induced by high glucose in PTEC and renal fibroblasts, cells that contribute importantly to tubulointerstitial fibrosis. In Figure 1, A and B, biotinylation of cell surface proteins showed increased csGRP78 in response to high glucose in both PTEC and renal fibroblasts, with surface expression increasing over time in renal fibroblasts but being most prominent at 6 hours in PTEC. In Figure 1, C and E, increased α2M production and the presence of extracellular α2M* in the medium with high glucose treatment were seen in PTEC. Parallel findings were observed in renal fibroblasts (Figure 1, D and F). The osmotic control mannitol did not increase surface expression of GRP78 on either PTEC or renal fibroblasts (Supplemental Figure 1, A and B; supplemental material available online with this article; https://doi.org/10.1172/jci.insight.183998DS1).

To determine whether csGRP78 and α2M* are relevant to tubulointerstitial fibrosis in vivo, we next assessed their expression in this compartment in 40-week-old type 1 diabetic Akita mice. We performed immunofluorescence on nonpermeabilized frozen kidney sections to identify cell surface rather than intracellular GRP78 and confirmed its surface localization using the cell surface marker wheat germ agglutinin (WGA) (11) (Supplemental Figure 2A). Colocalization with megalin further verified localization of csGRP78 and α2M* to PTEC (Supplemental Figure 2, B and C). Together, these data support specific upregulation of this receptor-ligand pair in disease.

We previously showed that in prostate cancer 1LN cells expressing high levels of csGRP78, α2M* interaction with csGRP78 induced a significant rise in intracellular calcium (12). We thus determined whether α2M* produced by PTEC and renal fibroblasts would induce a similar response. After PTEC (HK2 cells and primary human PTEC) or renal fibroblasts were treated for 48 hours with high glucose, conditioned medium was added to 1LN cells. As seen in Supplemental Figure 3, A–C, this increased intracellular calcium across all cell types. To confirm this was dependent on csGRP78/α2M* interaction, we tested the effects of adding an inhibitory peptide directly to 1LN cells exposed to PTEC or renal fibroblast media. This peptide is derived from the N-terminal Leu^91^-Leu^115^ sequence of GRP78, which binds to α2M* (13). Its effect on abolishing the calcium increase in the presence of PTEC or renal fibroblast media supports functional α2M* interaction with csGRP78.

We next evaluated whether inhibition of csGRP78 using the C-terminal targeting antibody C38, previously shown to attenuate α2M*-induced signaling through csGRP78 (14), would prevent high glucose–induced extracellular matrix production in PTEC and renal fibroblasts. In Figure 1, G and H, high glucose–induced production of fibronectin and collagen IV were inhibited by C38 in PTEC and renal fibroblasts, respectively. An IgG control had no effect on the response to high glucose in either cell type (Supplemental Figure 1, C and D). Further, we determined the effects of α2M* inhibition on high glucose pro-fibrotic responses to high glucose. Here, we used the antibody Fα2M, which recognizes the receptor binding domain of α2M* and which we previously showed neutralizes α2M* effects in 1LN cells (4). Figure 1, I and J, show that neutralizing α2M* prevented high glucose–induced fibronectin and collagen IV upregulation in both PTEC and renal fibroblasts. Similarly, the inhibitory peptide blocked high glucose–induced matrix protein upregulation (Figure 1, K and L). In parallel to these findings, inhibition of csGRP78, α2M*, or their interaction prevented pro-fibrotic signaling (measured as FAK and Akt activation) as well as production of matrix proteins fibronectin and collagen IV in human primary PTEC (Supplemental Figure 4, A and B). Together, these data support a potentially novel role for high glucose–induced csGRP78/α2M* pro-fibrotic signaling in kidney cells relevant to tubulointerstitial fibrosis as seen in the later stages of diabetic kidney disease.

### Inhibition of TGF-β1–induced csGRP78 or α2M* prevents matrix production by PTEC and renal fibroblasts.

**Figure 2:**
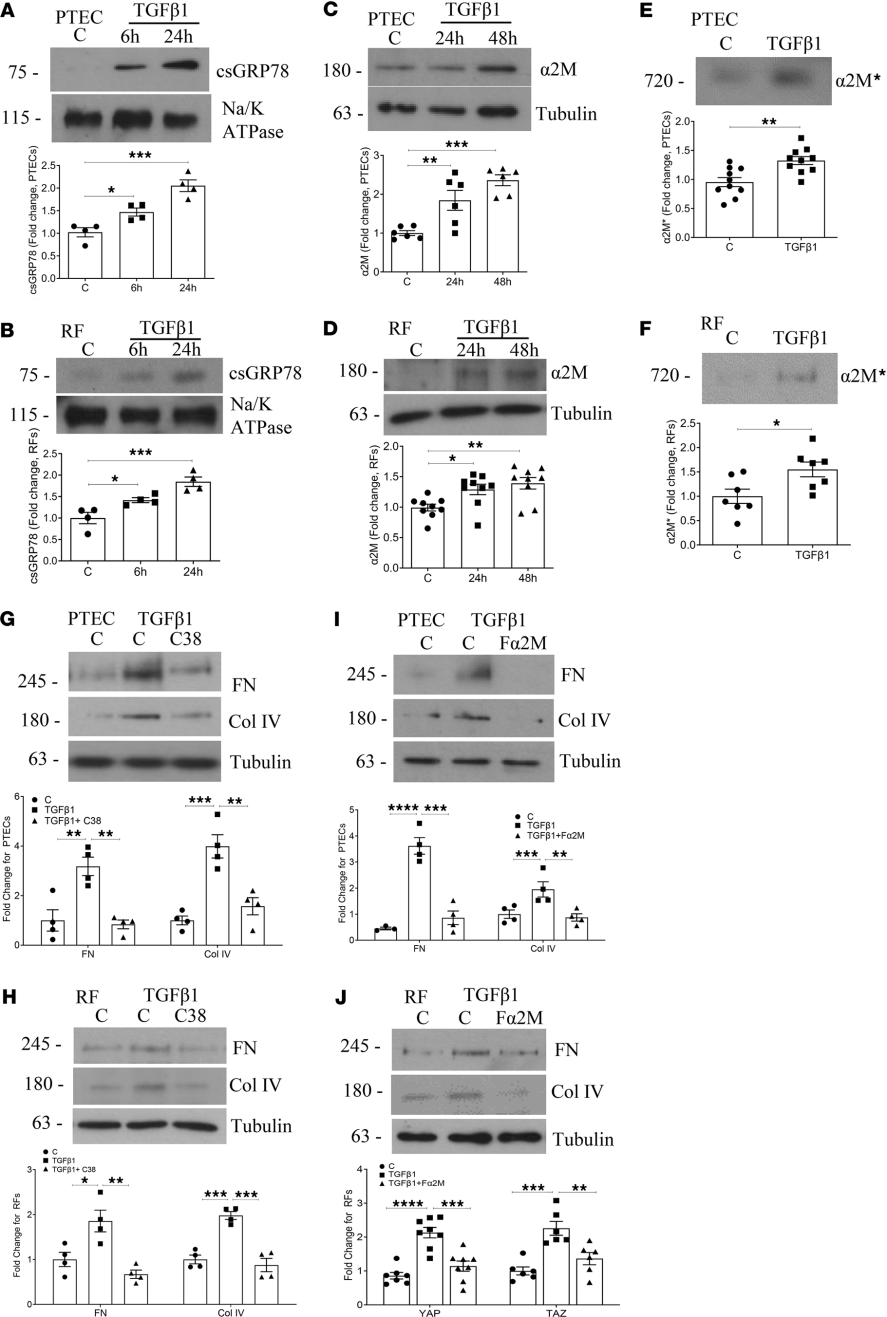
TGF-β1 induces csGRP78 localization and α2M activation in PTEC and renal fibroblasts. TGF-β1 (5 ng/mL, 6 or 24 hours) increased localization of GRP78 to the surface of both PTEC and renal fibroblasts, assessed by biotinylation (**A** and **B**, respectively) (*n* = 4). Similarly, TGF-β1– (5 ng/mL) induced α2M production (24 and 48 hours) and activation (48 hours) were increased in PTEC (*n* = 6 production and 10 activation) (**C** and **E**) and renal fibroblasts (*n* = 8–9 production and 7 activation) (**D** and **F**). TGF-β1– (5 ng/mL, 48 hours induced fibronectin and collagen IV production were attenuated by csGRP78 inhibition (**G** and **H** for PTEC and renal fibroblasts, respectively) (*n* = 4 for both). Similarly, α2M* inhibition in PTEC and renal fibroblasts prevented TGF-β1-induced matrix protein production (**I** and **J**) (5 ng/mL, 48 hours, *n* = 4 and 6) (*P < 0.05, **P < 0.01, ***P < 0.005; Kruskal-Wallis test used for α2M in **D**).

TGF-β1 is an established mediator of fibrosis in both diabetic and nondiabetic CKD (8, 15, 16). We have previously shown in mesangial cells that csGRP78/α2M* mediate TGF-β1 activation and pro-fibrotic signaling in response to high glucose through regulation of its nonproteolytic activator, thrombospondin-1 (6). However, whether csGRP78/α2M* can be directly increased by TGF-β1 is as yet unknown. To assess this, PTEC and renal fibroblasts were initially treated with TGF-β1 for various times. Biotinylation experiments to isolate cell surface proteins show an increase in csGRP78 at 6 and 24 hours in both cell types (Figure 2, A and B). Increased production and extracellular activation of α2M were also seen in both PTEC and renal fibroblasts at 48 hours (Figure 2, C–F). Thus, the translocation of GRP78 and upregulation, secretion, and activation of α2M can be elicited by TGF-β1 in addition to high glucose. We next assessed whether blocking this signaling pathway would also prevent TGF-β1–mediated matrix protein production. Inhibition of csGRP78 with the C38 antibody decreased expression of the matrix proteins fibronectin and collagen IV in PTEC (Figure 2G) and renal fibroblasts (Figure 2H). Inhibition of α2M* with Fα2M also attenuated matrix protein production in PTEC and renal fibroblasts (Figure 2, I and J). An IgG control had no effect on the TGF-β1 response in either cell type (Supplemental Figure 1, E and F). Hence, csGRP78/α2M* signaling is an important contributor to TGF-β1–mediated matrix production.

### csGRP78 and α2M* inhibition prevent high glucose–induced, but not TGF-β1–induced, Smad3 activation.

**Figure 3:**
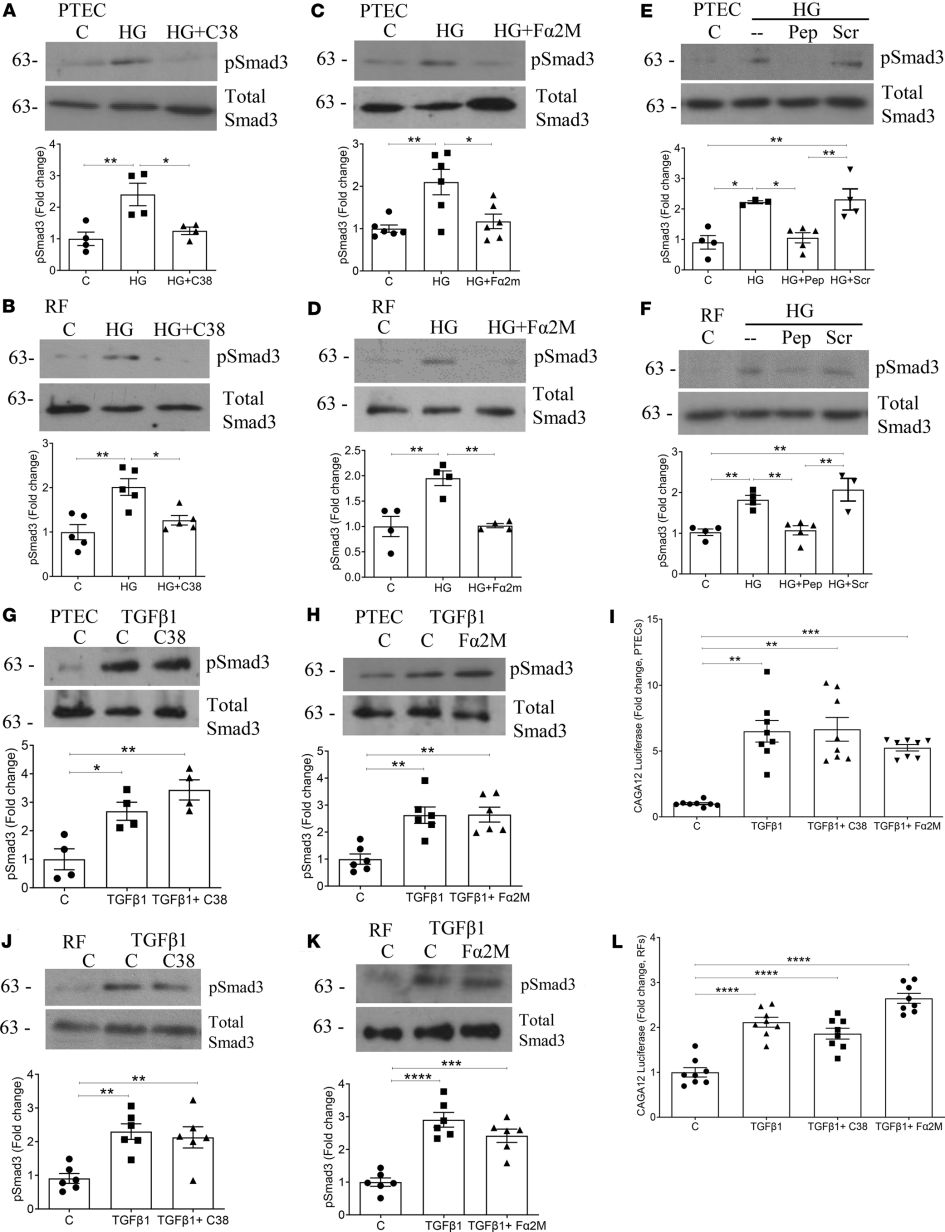
Smad3 activation by high glucose, but not by TGF-β1, is mediated by csGRP78/α2M*. High glucose– (30 mM, 48 hours) induced activation of Smad3 (measured as phosphorylation at Ser473/475) was prevented by csGRP78 inhibition in PTEC (*n* = 3–4) (**A**) and renal fibroblasts (*n* = 5) (**B**). Similarly, α2M* inhibition attenuated Smad3 activation by high glucose with either neutralizing antibody (*n* = 6 PTEC and 4 renal fibroblasts) (**C** = PTEC and **D** = renal fibroblasts) or inhibitory peptide (*n* = 4–5 PTEC and 3–4 renal fibroblasts) (**E** = PTEC and **F** = renal fibroblasts). In both PTEC and renal fibroblasts, csGRP78 (C38, 10 μg) did not prevent TGF-β1– (5 ng/mL, 48 hours) induced Smad3 activation (*n* = 4 and 6) (**G** and **J**, respectively). TGF-β1–induced Smad3 activation was also not prevented by α2M* inhibition (Fα2M, 10 μg) in PTEC or renal fibroblasts (*n* = 6 for both) (**H** and **K**, respectively). We confirmed these results using the Smad3-mediated reporter CAGA_12_-luciferase. TGF-β1-induced luciferase activation was not prevented by csGRP78 inhibition in either PTEC or renal fibroblasts (*n* = 8 for both) (**I** and **L**, respectively). Similarly, inhibition of α2M* did not prevent activation by TGF-β1 (**I** and **L**, respectively) (0.05 ng/mL, 24 hours, *n* = 8 for both) (*P < 0.05, **P < 0.01, ***P < 0.005, ****P < 0.0001; Kruskal-Wallis test used for CAGA_12_-luciferase in **K**).

We previously showed that csGRP78/α2M* inhibition prevents activation of Smad3, the canonical downstream mediator of TGF-β1 pro-fibrotic signaling, in response to high glucose in mesangial cells. Here we assessed whether this was also the case in PTEC and renal fibroblasts. Smad3 activation was assessed by its activating C-terminal phosphorylation (Ser473/475). In Figure 3, A and B, high glucose induced Smad3 activation in PTEC and renal fibroblasts, and this was inhibited by the C38 neutralizing antibody for csGRP78. Both the α2M* neutralizing antibody (Figure 3, C and D) and inhibitory peptide (Figure 3, E and F) also prevented high glucose–induced Smad3 activation in both cell types. This supports an important role for csGRP78/α2M* signaling in Smad3 activation across multiple cell types in response to high glucose.

Interestingly, in contrast with our findings above, TGF-β1–induced Smad3 phosphorylation was unaffected by csGRP78 or α2M* inhibition in PTEC (Figure 3, G and H). Lack of C38 or Fα2M effect on Smad3 transcriptional activity was confirmed using the reporter CAGA_12_-luciferase (Figure 3I). Similarly, inhibition of csGRP78 or α2M* did not prevent Smad3 activation by TGF-β1 in renal fibroblasts (Figure 3, J and K) or downstream activity of CAGA_12_-luciferase (Figure 3L). Since TGF-β1–induced pro-fibrotic effects were prevented by csGRP78 or α2M* inhibition, these data suggest a potential role for csGRP78/α2M* in regulating noncanonical TGF-β1 signaling.

### csGRP78/α2M* mediate noncanonical TGF-β1 signaling through YAP and TAZ.

**Figure 4:**
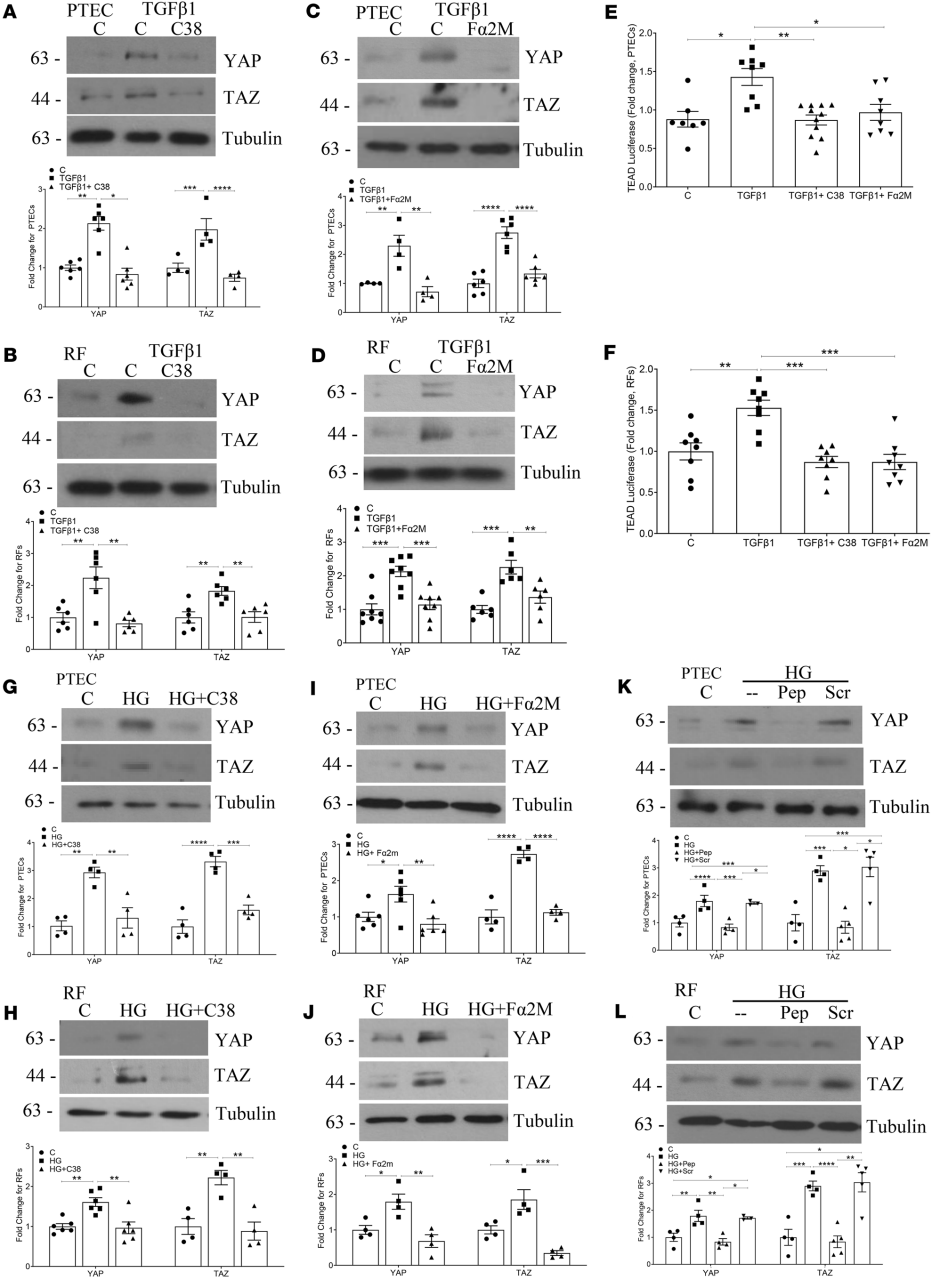
Noncanonical TGF-β1 mediators YAP and TAZ are regulated by csGRP78/α2M* in response to both high glucose and TGF-β1. Increased YAP and TAZ in response to TGF-β1 (5 ng/mL, 48 hours) were prevented by inhibition of csGRP78 (*n* = 4–6 PTEC and 6 renal fibroblasts) (**A** = PTEC and **B** = renal fibroblasts) and α2M* (*n* = 4–6 PTEC and 6–8 renal fibroblasts) (**C** = PTEC and **D** = renal fibroblasts). Using the TEAD-luciferase reporter construct, we confirmed that inhibition of both csGRP78 and α2M* in PTEC (*n* = 8–10) (**E**) and renal fibroblasts (*n* = 7–8) (**F**) prevented YAP/TAZ signaling in response to TGF-β1. High glucose– (30 mM, 48 hours) induced YAP and TAZ expression were also attenuated by csGRP78 (*n* = 4 PTEC and 4–6 renal fibroblasts) (**G** = PTEC and **H** = renal fibroblasts) and α2M* (*n* = 4–6 PTEC and 4 renal fibroblasts) (**I** = PTEC and **J** = renal fibroblasts) inhibition, as well as by the peptide inhibitor of csGRP78/α2M* interaction (*n* = 3–4, PTEC, **K**; and *n* = 4, renal fibroblasts, **L**). (*P < 0.05, **P < 0.01, ***P < 0.005, ****P < 0.0001.)

The known noncanonical TGF-β1 signaling mediators and transcriptional co-activators yes-associated protein 1 (YAP) and transcriptional co-activator with PDZ-binding motif (TAZ) have previously been shown to play a role in promoting the pro-fibrotic phenotype seen in CKD (17–20). They were also shown to be regulated by csGRP78 in pancreatic cancer (21). We thus assessed their potential role as factors downstream of csGRP78/α2M* in mediating the pro-fibrotic response to TGF-β1 as well as high glucose in PTEC and renal fibroblasts. In Figure 4, A and B, TGF-β1 increased expression of YAP and TAZ, and this was prevented by C38 inhibition of csGRP78 in both PTEC and renal fibroblasts, respectively. This was also seen with neutralization of α2M* (Figure 4, C and D). We next assessed functional effects on YAP/TAZ activity using the reporter transcriptional enhanced associate domain–luciferase (TEAD-luciferase). Here, inhibition of csGRP78 by C38 or α2M* by Fα2M prevented TGF-β1–induced TEAD activity in both PTEC (Figure 4E) and renal fibroblasts (Figure 4F). We observed similar effects with high glucose. In Figure 4, G and H, csGRP78 inhibition attenuated high glucose–induced expression of YAP and TAZ in PTEC and renal fibroblasts. Similar inhibition was seen with α2M* inhibition using either the neutralizing antibody Fα2M (Figure 4, I and J) or the inhibitory peptide (Figure 4, K and L). Thus, both TGF-β1– and high glucose–induced YAP/TAZ pro-fibrotic signaling is mediated by csGRP78/α2M* in PTEC and renal fibroblasts.

### Integrin β_1_ is required for mediating csGRP78 signaling.

**Figure 5:**
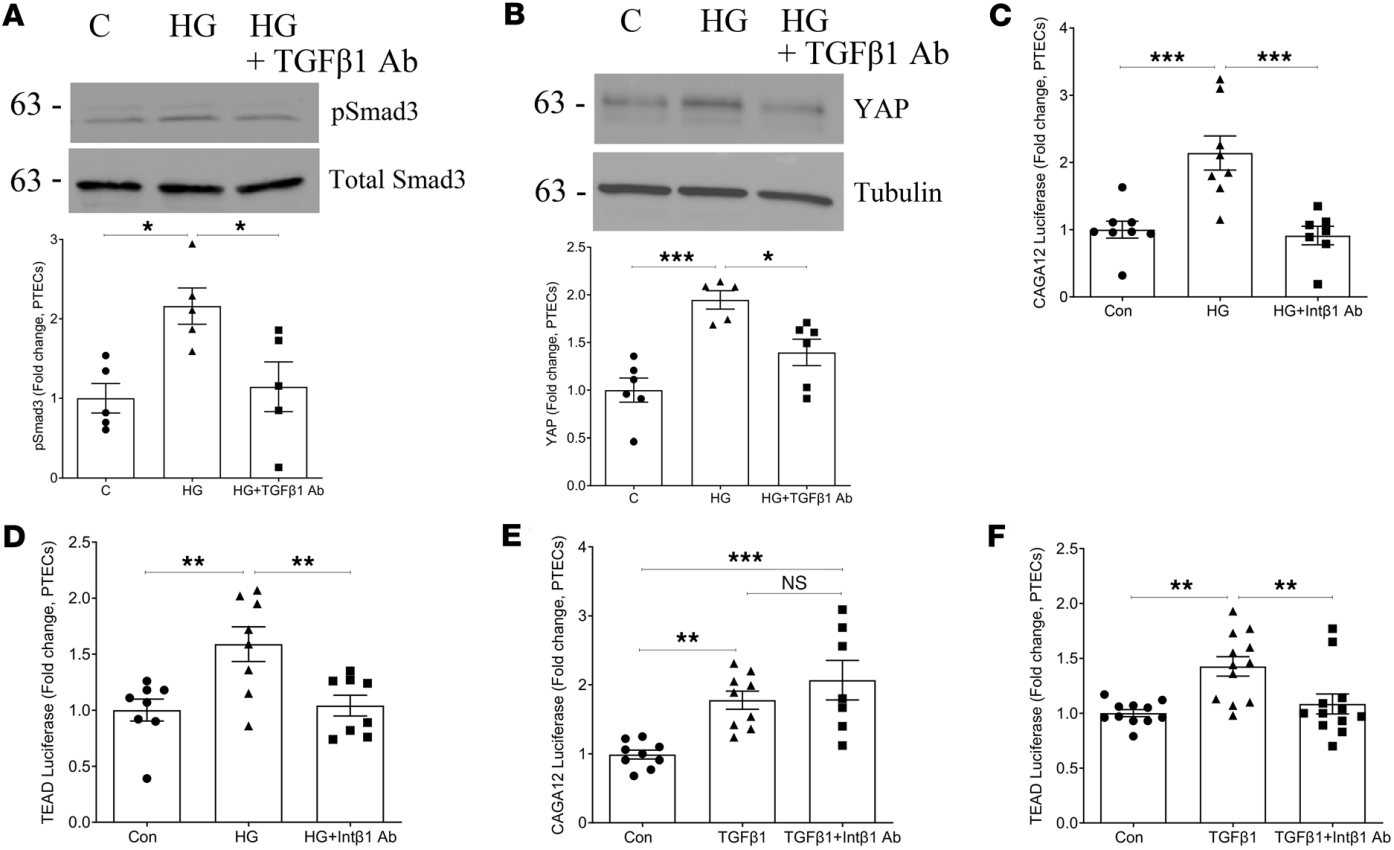
Requirement of integrin β_1_ for high glucose– and TGF-β1–induced YAP but not Smad3 activation. (**A**) High glucose– (30 mM, 48 hours) induced Smad3 activation (measured by its phosphorylation at S473/475) and (**B**) YAP upregulation were prevented by a TGF-β1 neutralizing antibody (10 μg) (*n* = 5 and *n* = 6 respectively). High glucose–induced activation of (**C**) CAGA_12_-luciferase, a measure of Smad3 transcriptional activity, and (**D**) TEAD-luciferase, a measure of YAP/TAZ activity, were prevented by an active integrin β_1_ neutralizing antibody (10 μg) (*n* = 7–8 and *n* = 8, respectively). TGF-β1– (0.05 ng, 24 hours) induced activation of (**E**) CAGA_12_-luciferase was not prevented by active integrin β_1_ neutralization, but (**F**) TEAD-luciferase activity was inhibited (*n* = 7–9 and *n* = 12, respectively). *P < 0.05, **P < 0.01, ***P < 0.005, ****P < 0.0001.

We sought to understand the basis for the differences in GRP78 involvement in signaling between high glucose and TGF-β1. We previously identified that integrin β_1_ is required for csGRP78 signaling and downstream upregulation of TGF-β1 synthesis, secretion, and signaling in response to high glucose in mesangial cells (5). We further implicated a role for csGRP78/α2M* in the activation of TGF-β1 through the regulation of its nonproteolytic activator, thrombospondin-1 (6). We thus hypothesized that integrin β_1_ may be required for csGRP78/α2M* modulation of TGF-β1 activation and activity in the setting of high glucose, whereas exogenous administration of active TGF-β1 would bypass this mechanism, thus giving rise to the differences in canonical signaling we observed in our in vitro studies. We first verified that Smad3 and YAP/TAZ activation by high glucose were indeed modulated by TGF-β1 in PTEC. Figure 5, A and B, show that TGF-β1 neutralization prevented both Smad3 phosphorylation and YAP upregulation by high glucose. We next confirmed that as for mesangial cells, integrin β_1_ was required for high glucose–induced Smad3 and YAP/TAZ activation. As seen in Figure 5, C and D, neutralization of integrin β_1_ prevented Smad3 activation, as measured by CAGA_12_-luciferase activity, and YAP/TAZ activation, measured by TEAD-luciferase activity. Conversely, TGF-β1–induced Smad3 transcriptional activation was not affected by integrin β_1_ neutralization, but YAP/TAZ activation was inhibited (Figure 5, E and F). These data show that in response to TGF-β1, YAP/TAZ but not Smad3 activation requires integrin β_1_.

### Inhibition of csGRP78, α2M*, or their interaction attenuates fibrosis in a mouse model of diabetic kidney disease.

We next sought to assess whether csGRP78 and α2M* expression were increased in type 1 diabetic Akita mice overexpressing TGF-β1. We previously showed worsened kidney pathology, particularly tubulointerstitial fibrosis, in this model compared with Akita mice without TGF-β1 overexpression in which tubulointerstitial fibrosis development is limited (22). Nondiabetic TGF-β1–overexpressing mice were used as controls (referred to as HH for TGF-β1 overexpression alone or HH-A for overexpression in Akita mice). csGRP78 was assessed by immunofluorescence of frozen kidney sections, with antibody labeling prior to fixation and permeabilization as described in Methods. WGA was used as a membrane marker. Supplemental Figure 5A shows increased staining for csGRP78 in both glomeruli and the tubulointerstitium, the latter highlighted by the colocalization mask shown in white, in HH-A mice at 20 and 30 weeks of age. Increased duration of diabetes augmented csGRP78, with no significant staining seen in 12-week-old mice. We also observed increased α2M* expression by immunofluorescence in HH-A mice at 20 and 30 weeks of age in both glomeruli and the tubulointerstitium (Supplemental Figure 5B). Interestingly, this was also increased in HH nondiabetic mice, though to a much lower degree than that seen in the diabetic mice. At 12 weeks of age, no significant staining was seen, similarly to that observed for csGRP78.

**Figure 6:**
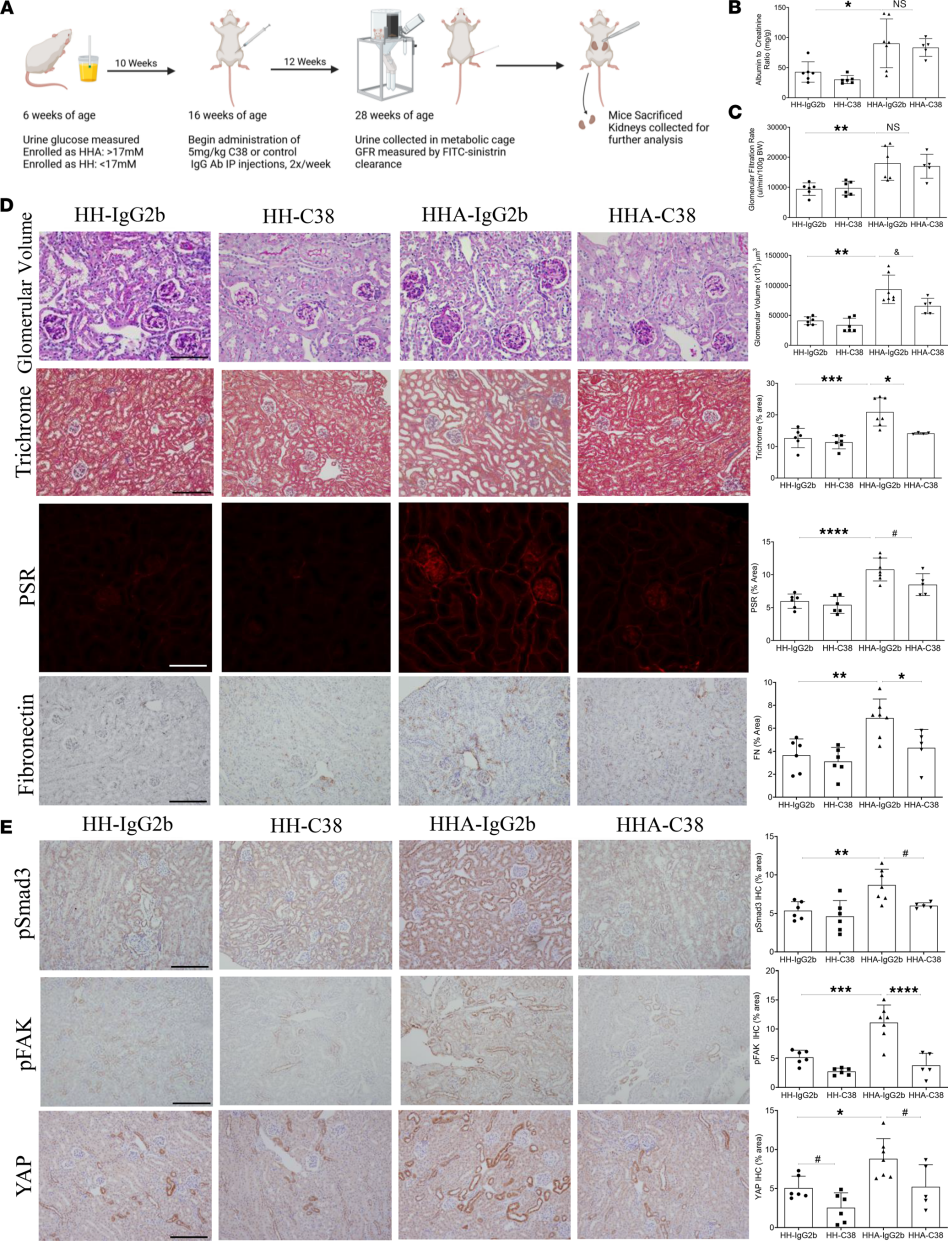
A csGRP78 neutralizing antibody attenuates fibrotic signaling in a mouse model of diabetic kidney disease. (**A**) Schematic outline of the study. Created in BioRender. Trink, J. (2025) https://BioRender.com/r0khlge FITC, fluorescein isothiocyanate; IP, intraperitoneal. The C38 csGRP78 antibody did not attenuate albuminuria, measured as the albumin-to-creatinine ratio (ACR) (**B**), the elevation in glomerular filtration rate (GFR) (**C**), or glomerular hypertrophy in diabetic mice (**D**) glomerular hypertrophy was reduced by C38 in diabetic mice. Fibrosis, as measured by trichome, Picrosirius red (PSR), and fibronectin, was reduced by C38 (**D**). Pro-fibrotic signaling, measured by Smad3 and FAK activation (phosphorylation at S473/475 and Y397, respectively), and YAP expression were all significantly reduced by C38 in diabetic mice (**E**) (*n* = 5–7, *P < 0.05, **P < 0.01, ***P < 0.005, ****P < 0.0001; ^#^*P* < 0.05 indicates significant only by *t* test; Kruskal-Wallis test used for glomerular volume in **D**; ^&^*P* < 0.05 indicates significant only by Mann-Whitney test. Scale bar represents 20 μm in all panels except **D**, glomerular volume, where scale bar represents 10 μm.

Given this time course, we began treatment studies of csGRP78 or α2M* inhibition at 16 weeks of age, prior to established increases in both receptor and ligand. Mice were followed for 12 weeks until 28 weeks of age. In the first study, mice received 2 injections weekly of C38 or an isotype control (IgG2b) antibody dosed at 5 mg/kg. Figure 6A shows a timeline of this study. After 12 weeks of C38 or IgG2b administration, we observed increased albuminuria, measured by ACR (Figure 6B), and hyperfiltration, measured by the glomerular filtration rate (GFR) (Figure 6C) in all HH-A mice. These were unaffected by C38 treatment. Interestingly, although kidney hypertrophy was unaffected (Supplemental Figure 6A), we did observe a significant decrease in glomerular volume of HH-A mice treated with C38 compared with HH-A controls (Figure 6D). Compared with HH-A mice treated with control IgG2b antibody, C38-treated HH-A mice had significantly less fibronectin and collagen expression (measured by trichrome and PSR staining) (Figure 6D). We next assessed proteins downstream of csGRP78/α2M* we have shown regulate pro-fibrotic signaling in vitro (3–5). Here, we observed a significant decrease in Smad3 and FAK activation (measured by their phosphorylation at S473/475 and Y397, respectively), as well as in YAP expression with csGRP78 inhibition in HH-A mice (Figure 6E). Thus, inhibition of csGRP78 by C38 protected against the development of fibrosis in diabetic kidney disease.

**Figure 7:**
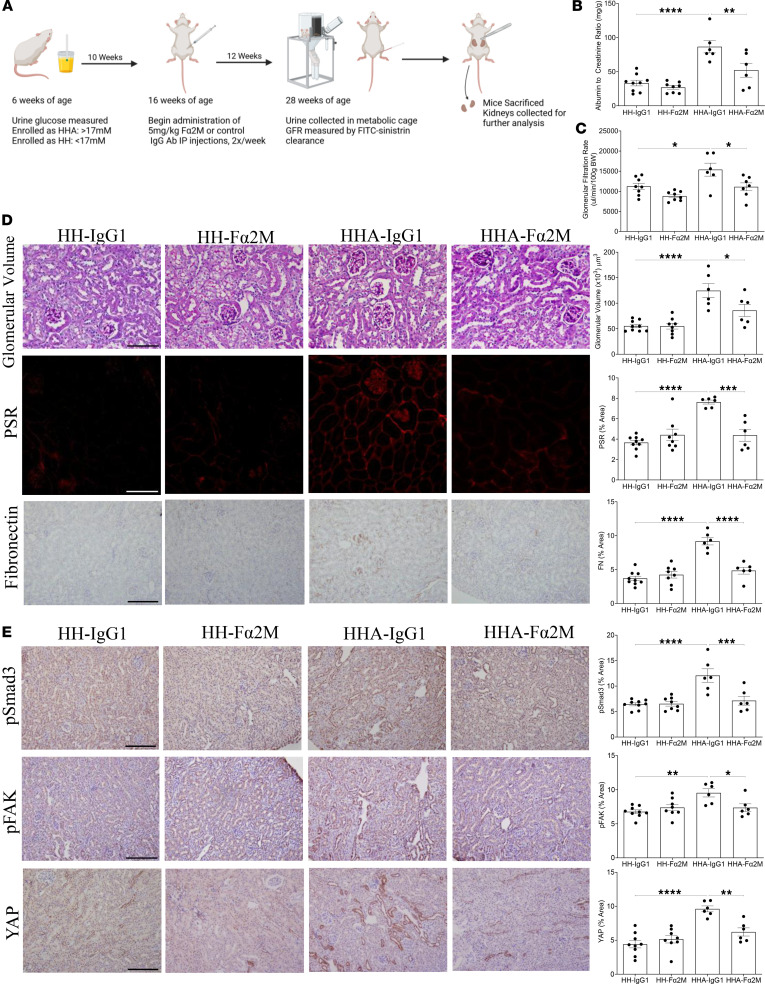
Inhibition of α2M* with the Fα2M neutralizing antibody attenuates fibrotic signaling in a mouse model of diabetic kidney disease. (**A**) Schematic outline of the study. Created in BioRender. Trink, J. (2025) https://BioRender.com/k0jedtz Fα2M reduced albuminuria, measured as the albumin-to-creatinine ratio (ACR) (**B**), the elevation in glomerular filtration rate (GFR) (**C**), and glomerular hypertrophy in diabetic mice (**D**). Fibrosis, as measured by PSR and fibronectin, was also significantly reduced by Fα2M (**D**). Activation of pro-fibrotic signaling, measured by Smad3 (phospho-S473/475) and FAK (phospho-Y397), as well as YAP expression, were significantly reduced in diabetic mice treated with Fα2M (**E**) (*n* = 6–9; *P < 0.05, **P < 0.01, ***P < 0.005, ****P < 0.0001; scale bar represents 20 μm in all panels except **D**, glomerular volume, where scale bar represents 10 μm).

We next assessed whether inhibition of α2M* by using the Fα2M antibody would show similar antifibrotic effects in vivo. Confirmation of this antibody’s ability to bind to α2M* was assessed by surface plasmon resonance (Supplemental Figure 7). An outline of this study is depicted in Figure 7A. After 12 weeks of treatment with Fα2M or an isotype control (IgG1), dosed as for the C38 study at 5 mg/kg twice weekly, we observed a significant decrease in ACR (Figure 7B), GFR (Figure 7C), kidney hypertrophy (Supplemental Figure 6B), and glomerular hypertrophy (Figure 7D). Further, kidney fibrosis, measured by PSR (collagens I/III) and fibronectin, was also significantly decreased in HH-A Fα2M-treated mice compared with mice given isotype control antibody (Figure 7D). Last, the pro-fibrotic signaling proteins Smad3 pS473/475, FAK pY397, and YAP were all decreased by α2M* inhibition in HH-A mice (Figure 7E). These data support α2M* inhibition as an antifibrotic agent for diabetic kidney disease.

**Figure 8:**
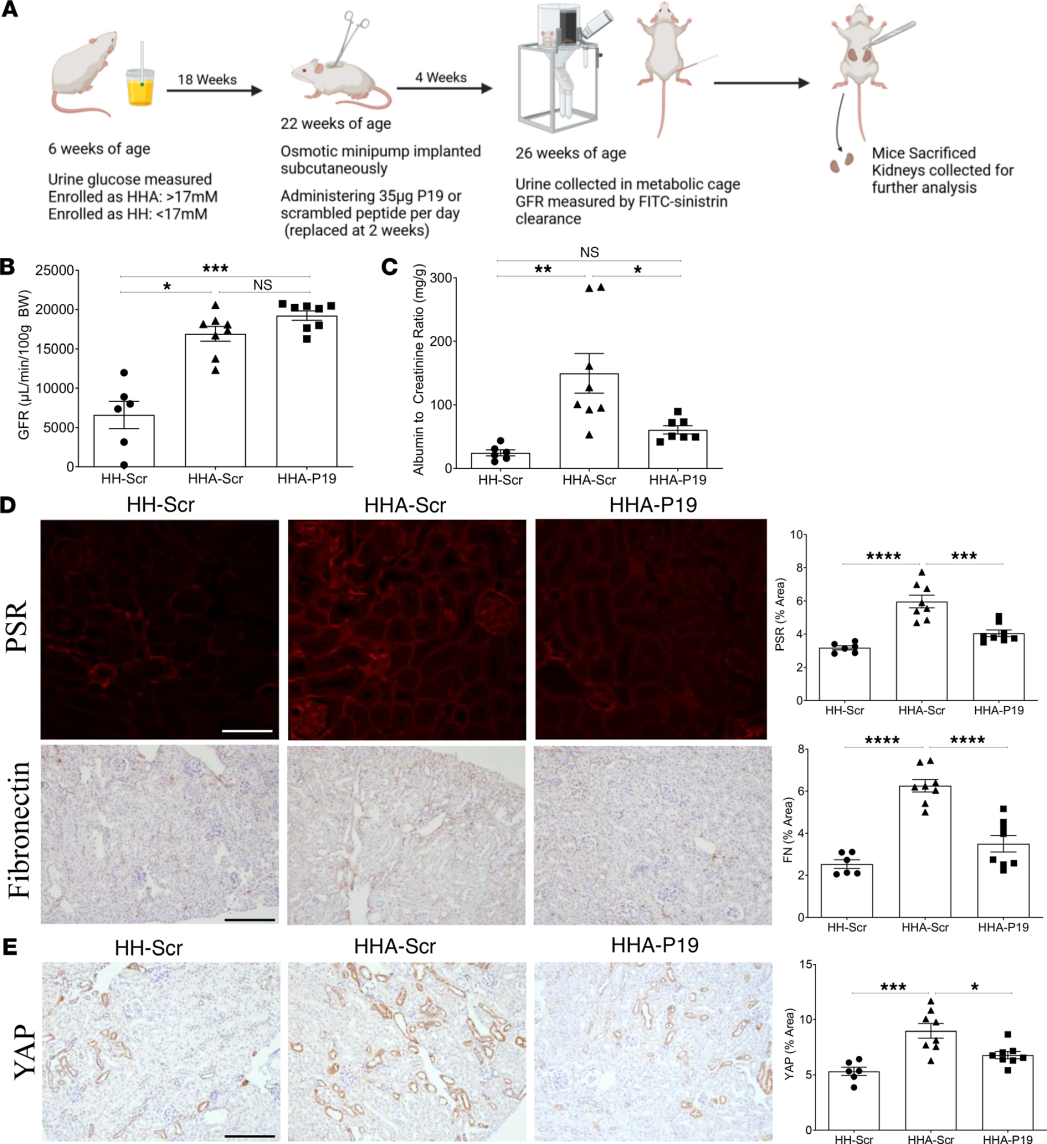
Inhibition of csGRP78/α2M* interaction by inhibitory peptide reduces fibrosis in a mouse model of diabetic kidney disease. (**A**) Schematic outline of the study. Created in BioRender. Trink, J. (2025) https://BioRender.com/untls0u (**B**) Active peptide treatment did not prevent the elevation in glomerular filtration rate (GFR) in diabetic mice. (**C**) Active peptide reduced albuminuria in diabetic mice, measured as the albumin-to-creatinine ratio (ACR). (**D**) Fibrosis, as measured by PSR and fibronectin, was significantly reduced by inhibitory peptide. (**E**) Activation of pro-fibrotic signaling, measured by YAP expression, was reduced by active peptide (*n* = 6–8, *P < 0.05, **P < 0.01, ***P < 0.005, ****P < 0.0001; scale bar represents 20 μm).

Last, we assessed whether inhibition of csGRP78/α2M* interaction using our inhibitory peptide would prevent fibrosis in diabetic kidney disease. The study outline is shown in Figure 8A. After 4 weeks of active or scrambled control peptide administration (35 μg/d) via osmotic minipump, there was no difference in GFR (Figure 8B) or kidney hypertrophy (Supplemental Figure 6C). In comparison with treatment with Fα2M, this is likely due to the much shorter treatment time with peptide. However, ACR (Figure 8C) and kidney fibrosis, measured by PSR and fibronectin (Figure 8D), were significantly decreased by active peptide in diabetic mice. Further, YAP was also decreased by inhibitory peptide but not by scrambled control in diabetic mice (Figure 8E). Together, these studies support the therapeutic potential of inhibiting csGRP78/α2M* signaling as an antifibrotic target in diabetic kidney disease.

### Inhibition of α2M* attenuates fibrosis after unilateral ureteral obstruction.

To evaluate the potential role of csGRP78/α2M*-mediated pro-fibrotic signaling in a nondiabetic model of kidney disease, we first determined their expression after unilateral ureteral obstruction (UUO), a model characterized by tubulointerstitial fibrosis and inflammation (23). In Supplemental Figure 8A, csGRP78 (colocalized with plasma membrane WGA) was increased at 7, 14, and 21 days after UUO, with a small increase seen as early as 1 day following model creation. Similarly, α2M* was also increased at these time points (Supplemental Figure 8B). We further confirmed csGRP78 and α2M* colocalization with PTEC in the model at 10 days after UUO, our time point for assessment of therapeutic intervention (Supplemental Figure 9, A and B). These data support a potential role for csGRP78/α2M* in mediating tubulointerstitial fibrosis in this model.

**Figure 9:**
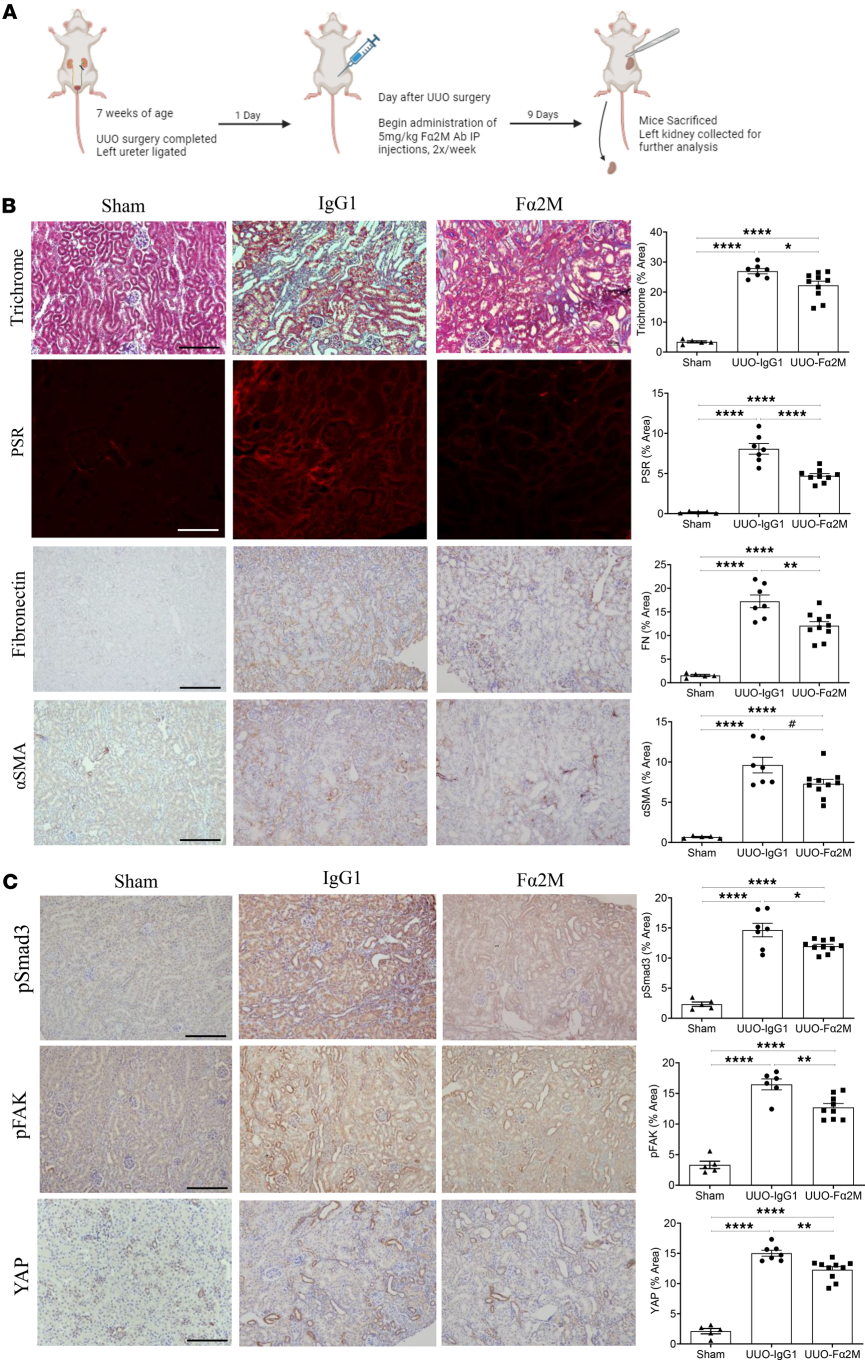
Inhibition of α2M* with the Fα2M neutralizing antibody reduces fibrosis in the UUO model of CKD. (**A**) Schematic outline of the study. Created in BioRender. Trink, J. (2025) https://BioRender.com/2rod2yk (**B**) Fα2M significantly reduced fibrosis measured as trichrome, PSR, and fibronectin, as well as the myofibroblast marker α–smooth muscle actin (α-SMA). (**C**) Treatment with Fα2M also prevented Smad3 and FAK activation (measured by phosphorylation at S473/475 and Y397, respectively), as well as increased YAP expression compared with control IgG1 (*n* = 7–9; ^*^P < 0.05, ^**^P < 0.01, ****P < 0.0001; ^#^*P* < 0.05 significant by *t* test; scale bar represents 20 μm).

We next tested the effects of the Fα2M antibody or its isotype control (IgG1) antibody at 5 mg/kg beginning the day after UUO surgery, with additional injections occurring on the fourth and eighth day prior to harvest 10 days after UUO creation. Figure 9A depicts the outline of this study. Figure 9B shows that fibrosis, assessed by trichome, PSR, and fibronectin as well as the marker of activated fibroblasts, α–smooth muscle actin (α-SMA) (24), were all significantly attenuated by Fα2M.

TGF-β1 activity has been shown to play an important role in fibrosis induced by UUO (25, 26), with inhibition of either its Smad-dependent or -independent signaling reducing tubulointerstitial fibrosis (27–29). Figure 9C shows that activation of Smad3 and FAK and upregulation of YAP were all significantly decreased by Fα2M. Interestingly, the observed decrease in Smad3 activation in vivo did not align with our in vitro findings, suggesting that other factors such as mechanical stress may be important to Smad3 activation and regulated by α2M* signaling. These data support a potentially novel role for α2M* inhibition in preventing the fibrotic phenotype in CKD.

### Peptide inhibition of csGRP78/α2M* interaction attenuates pro-fibrotic signaling and fibrosis in UUO.

**Figure 10:**
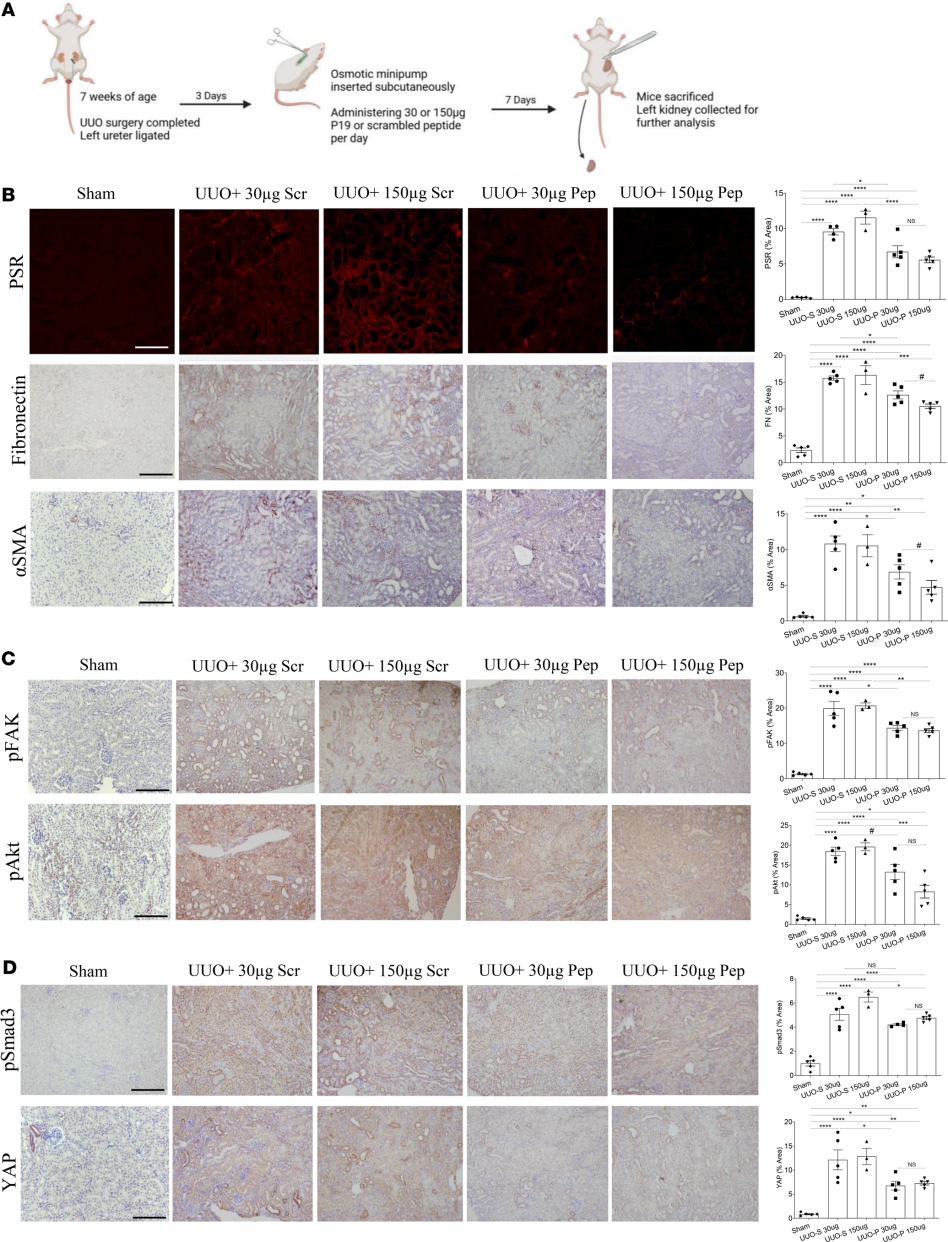
Treatment with a peptide inhibitor of csGRP78/α2M* interaction reduces fibrosis in the UUO model. (**A**) Schematic outline of the study. Created in BioRender. Trink, J. (2025) https://BioRender.com/8b94uo6 Treatment was initiated 3 days after UUO. (**B**) Active, but not scrambled, peptide dose-dependently inhibited fibrosis as measured by staining for PSR and fibronectin, as well as α-SMA, a marker for myofibroblast activation. (**C**) Pro-fibrotic signaling through FAK and Akt activation downstream of csGRP78/α2M*, assessed by their phosphorylation (Y397 and S473/475, respectively), was decreased by peptide. Akt phosphorylation showed dose dependency. (**D**) Smad3 activation (phospho-S473/475) and increased YAP were both reduced by active, but not scrambled, peptide (*n* = 3–5; *P < 0.05, **P < 0.01, ***P < 0.001, ****P < 0.0001; ^#^*P* < 0.05 indicates significant only by *t* test; scale bar represents 20 μm).

We next wished to test the in vivo efficacy of our peptide inhibitor of csGRP78/α2M* interaction in a delayed treatment model. Mice were treated starting at day 3 after UUO creation with inhibitory or scrambled peptide at either a low (30 μg/d) or a high (150 μg/d) dose via osmotic minipump for 7 days. An outline of this study is described in Figure 10A. As seen in Figure 10B, fibrosis, assessed by PSR and fibronectin immunohistochemistry, was dose-dependently attenuated by the active peptide. Activation of myofibroblasts, measured by α-SMA, was also dose-dependently decreased (Figure 10B). We next assessed FAK and Akt, important mediators of UUO-induced fibrosis (30), which we previously showed were activated by csGRP78 in mesangial cells (3). The activation of both was also decreased by active peptide (Figure 10C), as were Smad3 activation and YAP expression (Figure 10D). Together, these data support a role for csGRP78/α2M* in regulating pro-fibrotic signaling and extracellular matrix production in the UUO model.

As recruitment of activated inflammatory cells was shown to contribute to the pathogenesis of kidney fibrosis, we next determined whether peptide inhibition could attenuate inflammation. In Supplemental Figure 10A, T cell infiltration was mildly affected by csGRP78/α2M* inhibition at the higher peptide dose. We also observed a decrease in macrophage infiltration with the higher peptide dose. These data implicate a potential role for csGRP78/α2M* in mediating inflammation that contributes to the fibrotic phenotype seen in kidney disease (31).

Last, low- and high-dose peptide administration also decreased α2M* expression (Supplemental Figure 10B), showing that peptide inhibition of this signaling pathway does not lead to α2M* accumulation. These data suggest that α2M* clearance through its lower affinity receptor low density lipoprotein receptor-related protein 1 is not impaired by peptide treatment (32, 33).

**Figure 11:**
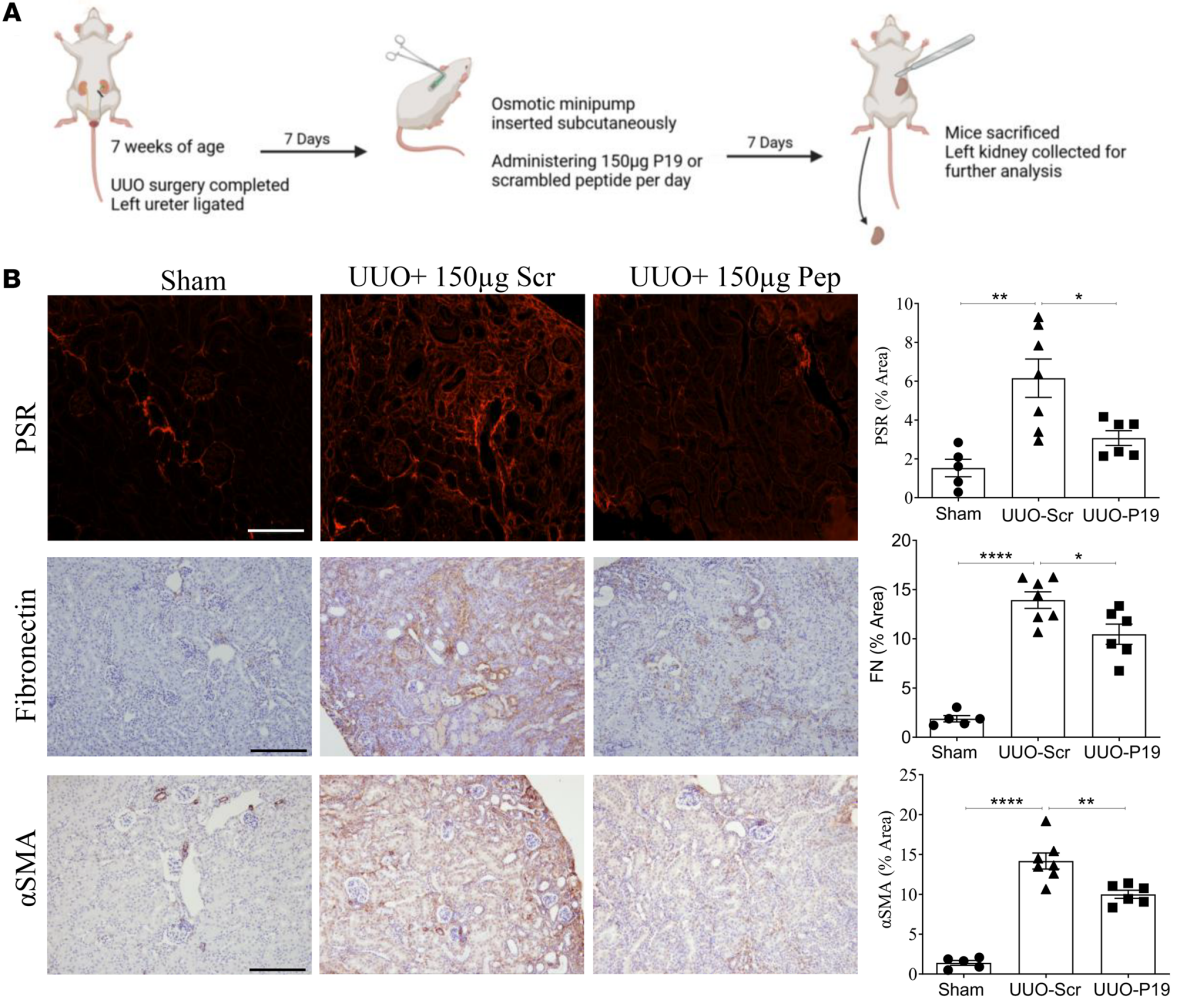
Delayed treatment with a peptide inhibitor of csGRP78/α2M* interaction reduces fibrosis in the UUO model. (**A**) Peptide administration was delayed until 7 days after UUO surgery. Created in BioRender. Trink, J. (2025) https://BioRender.com/0ul7cxg Mice were followed for an additional 7 days as shown in the schematic outline of this study. (**B**) Fibrosis, measured by PSR and fibronectin, as well as α-SMA, a marker for myofibroblast activation, were decreased by inhibitory peptide but not scrambled control peptide (*n* = 5–7; *P < 0.05, **P < 0.01, ****P < 0.0001; scale bar represents 20 μm).

Finally, we sought to test the antifibrotic effects of peptide treatment after more established fibrosis. We thus implanted osmotic minipumps with scrambled or active peptide 7 days after UUO creation and assessed kidneys at day 14, as outlined in the schematic in Figure 11A. As seen in Figure 11B, fibrosis as measured by PSR and fibronectin were markedly decreased in UUO treated with active compared with scrambled peptide. The increase in the fibroblast activation marker α-SMA was similarly decreased by active peptide (Figure 11B). These data support efficacy of csGRP78/α2M* signaling in the setting of established kidney fibrosis.

### α2M transcript levels are elevated in CKD from various causes.

**Figure 12:**
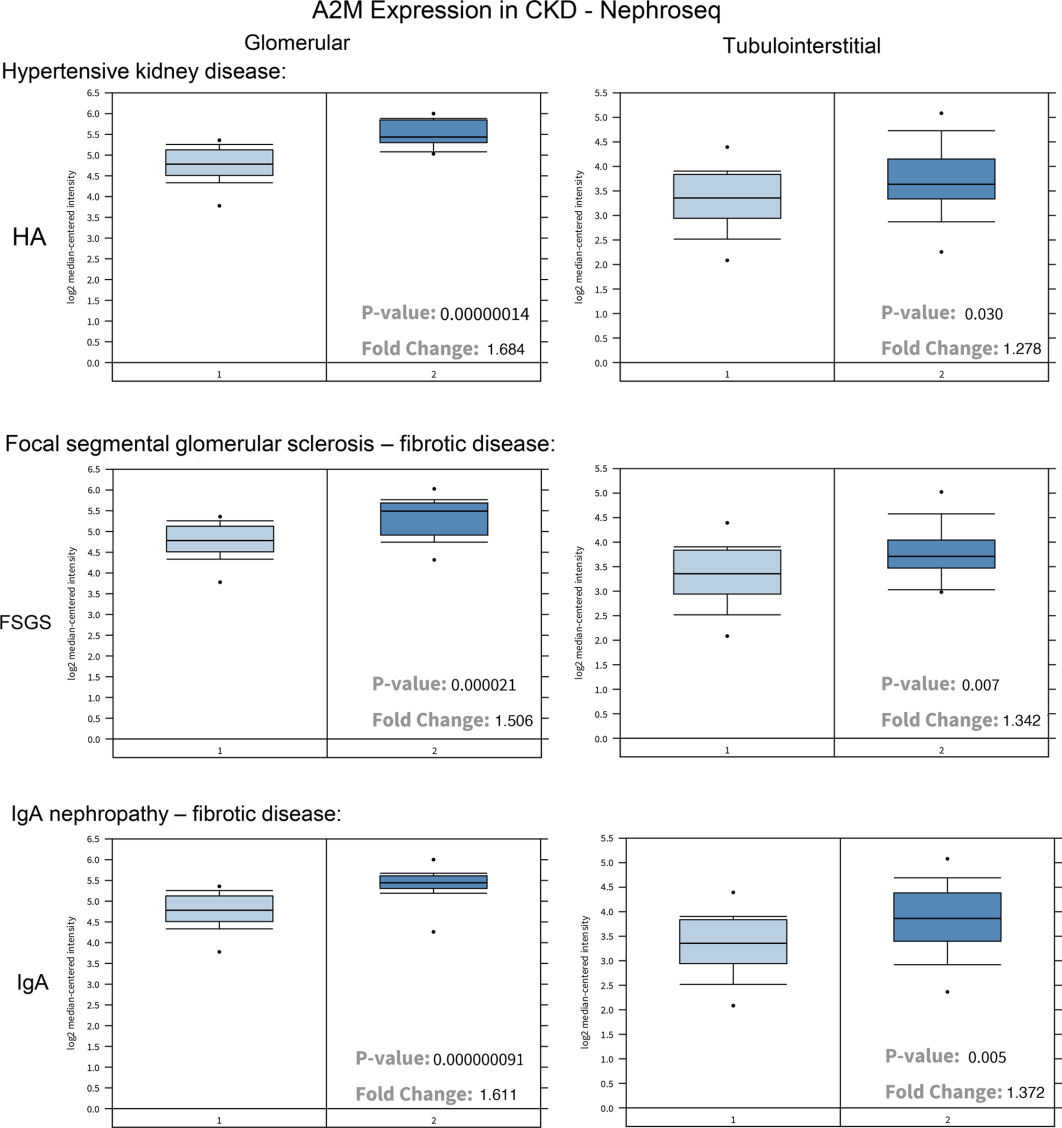
RNA-Seq data support a role for *α2M* transcript increases in human patients with CKD. Data available from the NephroSeq database (https://www.nephroseq.org/resource/login.html) were used. *α2M* RNA expression was significantly increased in kidneys in glomeruli and the tubulointerstitium from patients with various forms of CKD, including hypertensive CKD, FSGS, and IgA nephropathy, compared with healthy living donors.

Although both the cell surface localization of GRP78 and activation of α2M are posttranslational events, our data show that α2M production is increased upon exposure to either high glucose or TGF-β1. It is also increased progressively with disease duration in diabetic kidneys (4) and after UUO. Supporting local production, increased urinary α2M was recently identified in a discovery proteomics study in stages 3 and 4 of disease as a potential biomarker of progressive diabetic kidney disease (34). In our previous studies in mesangial cells, we identified the transcriptional upregulation of α2M and showed that increased transcript was also seen in human diabetic kidney disease in both the glomerular and tubulointerstitial compartments using NephroSeq data (6). Increased α2M transcript has additionally been identified in human nondiabetic CKD in mesangial cells and glomerular endothelial cells in focal glomerular sclerosis (FSGS), in which it was associated with disease progression and poor kidney prognosis (35). We thus assessed α2M transcript in additional kidney diagnoses that lead to fibrosis and CKD. In the NephroSeq dataset, we found increased α2M in FSGS as previously identified. We also found increased α2M in other CKD diagnoses characterized by progressive fibrosis, including hypertensive kidney disease and IgA nephropathy (Figure 12). Increased expression was observed in both the glomerular and tubulointerstitial compartments. These data support local upregulation and synthesis of α2M in various kidney diseases marked by fibrosis.

## Discussion

**Figure 13:**
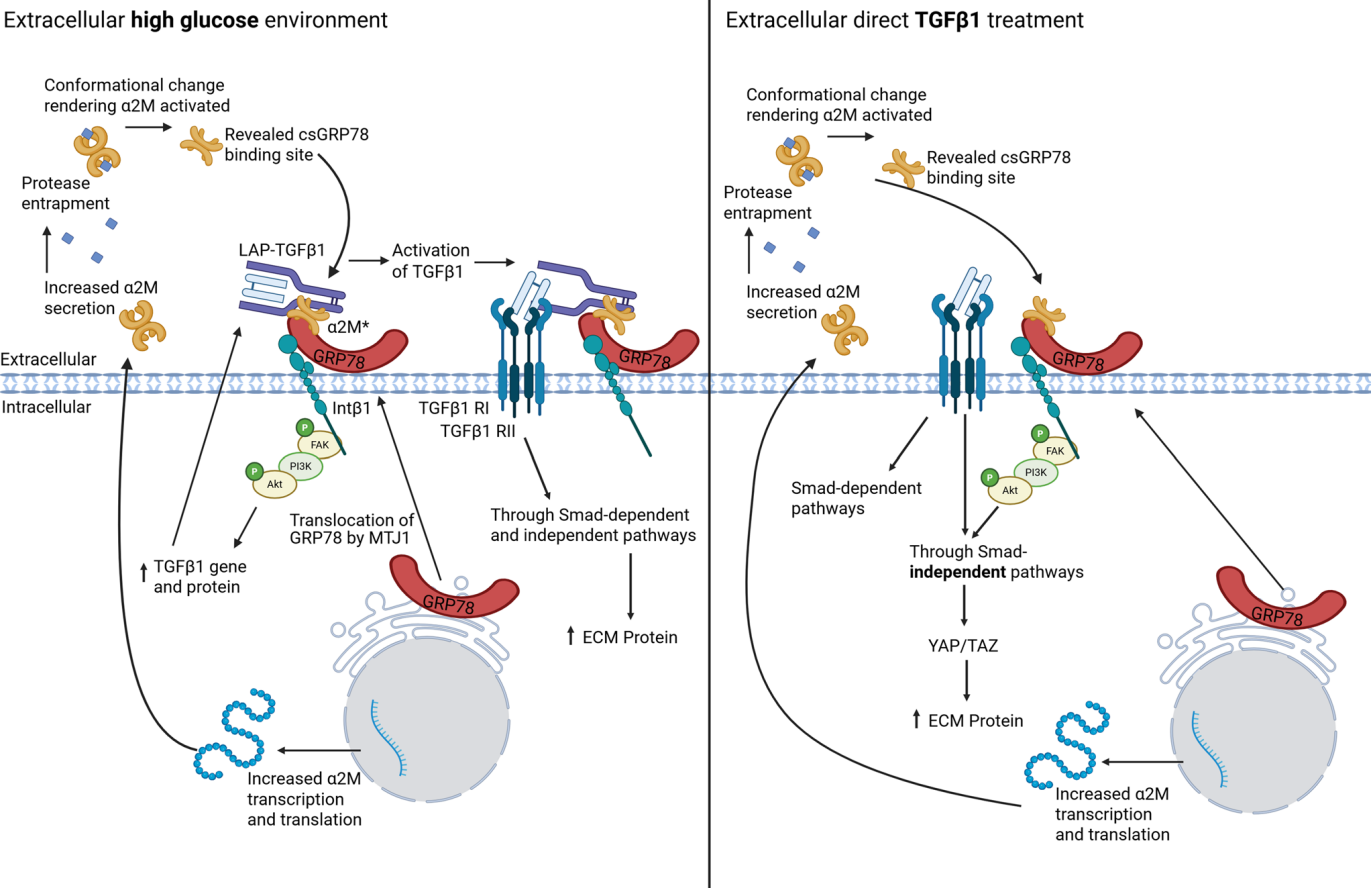
Summary of findings in the context of previous data. High glucose induces α2M production, secretion, and activation as well as translocation of GRP78 to the cell surface of mesangial cells, PTEC, and renal fibroblasts. Here α2M* binds to csGRP78, which is tethered by activated integrin β_1_, to activate pro-fibrotic FAK/PI3K/Akt signaling. This leads to increased TGF-β1 production, secretion, and activation to promote pro-fibrotic signaling through both canonical and noncanonical pathways, ultimately leading to increased matrix protein production. In the setting of direct active TGF-β1 treatment, α2M production and activation, as well as csGRP78 cell surface translocation, are induced in cells. However, this mechanism only mediates noncanonical TGF-β1 signaling, which requires integrin β_1_. Created in BioRender. Trink, J. (2025) https://BioRender.com/p8navir

In this study, we extend the relevance of csGRP78/α2M* pro-fibrotic signaling to tubulointerstitial fibrosis, characteristic of CKD progression regardless of underlying etiology and an important predictor of kidney failure (8, 9, 36, 37). We show the presence of csGRP78 and α2M* in the tubulointerstitium of diabetic kidneys. In response to high glucose, csGRP78 and α2M* regulate pro-fibrotic signaling in both PTEC and renal fibroblasts, cell types key to the development of tubulointerstitial fibrosis. Together with our previous work showing that csGRP78/α2M* regulate pro-fibrotic responses to high glucose in mesangial cells (3, 4), these data support the importance of this signaling pair as important mediators of pathogenic high glucose responses across varied cell types in the diabetic kidney in both early and later stages of kidney fibrosis. This is summarized in the context of our previous data in Figure 13.

Our previous studies have shown that the regulation of fibrosis by csGRP78/α2M* is at least in part due to their promotion of TGF-β1 production, activation, and downstream pro-fibrotic signaling in response to high glucose (5, 6). Although TGF-β1 is an important pathologic regulator of fibrosis in kidney disease (15), its pleiotropic functions limit its direct inhibition. This was shown in clinical trials in which TGF-β1 neutralization had limited therapeutic benefit when dosed at levels that minimized severe adverse effects, resulting in early trial termination (7, 38). Thus, targeting aberrant TGF-β1 activity without its complete neutralization is of current therapeutic interest. We now demonstrate that csGRP78/α2M* are important in cellular pro-fibrotic responses to direct TGF-β1 stimulation. Thus, TGF-β1 itself induces GRP78 translocation to the cell surface and α2M upregulation and activation in PTEC and renal fibroblasts. Importantly, the prevention of TGF-β1–induced matrix production by csGRP78/α2M* inhibition suggested potential relevance to fibrosis in nondiabetic CKD. Indeed, we show a progressive and time-dependent increase in both csGRP78 and α2M* in UUO, a nondiabetic model marked by tubulointerstitial fibrosis. Furthermore, our previous study showed increased α2M* expression in glomeruli and the tubulointerstitium in the 5/6 nephrectomy hypertensive CKD mouse model (4), in which TGF-β1 is known to be a critical pathogenic mediator (39).

The de novo expression and extracellular localization of ligand and receptor and relevance across multiple kidney cell types render csGRP78/α2M* attractive therapeutic targets. Here we tested the therapeutic value of 3 distinct ways of disrupting the interaction between csGRP78 and α2M*, targeting either ligand or receptor, in both diabetic and nondiabetic models. All were effective in decreasing fibrosis. Importantly, in the UUO model, treatment was delayed until 3 and 7 days after model creation, when fibrosis is already well established. The observed dose-dependent efficacy of the inhibitory peptide in this model supports benefit in treatment of established disease, as would be seen in the clinic, and provides a strong foundation for further development of this target as an antifibrotic.

Interestingly, in these studies, we identify an important difference in the role of csGRP78/α2M* in the activation of Smad3, an important transducer of TGF-β1 pro-fibrotic effects. While csGRP78/α2M* regulated Smad3 activation under high glucose, TGF-β1–induced Smad3 activation did not require csGRP78 or α2M*. It is known that high glucose induces TGF-β1 production and Smad3-dependent pro-fibrotic signaling (5, 6, 40), as we also show in our current studies. Once secreted into the extracellular space, TGF-β1 resides in an inactive complex. We had previously identified that in high glucose, integrin β_1_ together with csGRP78 mediated TGF-β1 upregulation and its activation through increased production of thrombospondin-1 (6). We now verify this mechanism also occurs in PTEC, while direct treatment with active TGF-β1 bypasses this requirement. Interestingly, the observed decrease in Smad3 activation in vivo in the UUO model did not align with our in vitro findings, suggesting that other factors such as mechanical stress may be important to Smad3 activation and regulated by α2M* signaling in vivo.

Noncanonical TGF-β1 signaling mediators including YAP and TAZ contribute importantly to TGF-β1 pro-fibrotic effects. Basally, YAP and TAZ are excluded from the nucleus and undergo cytoplasmic proteasomal degradation after phosphorylation by kinases LATS1/2. Stimuli such as growth factors and mechanical stress reduce YAP/TAZ phosphorylation, allowing nuclear accumulation and upregulation of gene expression in conjunction with TEAD family transcription factors (9, 17). YAP/TAZ are known to contribute to fibrosis in both diabetic and nondiabetic CKD (17, 19, 41). Recently, YAP and TAZ were shown to be regulated by csGRP78 in pancreatic cancer cells (21). In vivo, their activation may also be increased by the mechanical stress resulting from tissue stiffening due to extracellular matrix accumulation (42). Our data now show that csGRP78/α2M* mediate YAP/TAZ activation by high glucose or TGF-β1 in several kidney cell types. However, in vivo we observed that csGRP78/α2M* inhibition prevented an increase in YAP but not TAZ expression. Although some studies have identified independent regulation of, and signaling by, YAP and TAZ (17, 41, 43), it is also possible that other stimuli present in vivo, which cannot be accounted for in an isolated cell culture environment, may have caused these differences.

The UUO model is characterized by marked inflammatory cell recruitment. Our data show that at the higher dose of peptide used, inhibition of csGRP78/α2M* interaction attenuated macrophage and T cell infiltration. The contribution of macrophages to kidney fibrosis is well established (44, 45). Macrophages are known to basally express csGRP78, and α2M* promotes their migration, phagocytosis, and intracellular signaling (46). It is thus possible that α2M* promotes the recruitment and/or activation of macrophages in CKD. Furthermore, TGF-β1/Smad3 signaling was shown to regulate transition of bone marrow–derived macrophages to myofibroblasts in the UUO kidney to promote fibrosis (44, 45). csGRP78/α2M* signaling may thus promote recruitment, activation, and phenotypic transition of macrophages to contribute to kidney fibrosis. Future studies would more precisely characterize the mechanisms by which csGRP78/α2M* regulate macrophages and possibly directly regulate T cells in the fibrotic kidney.

Limitations to this study include the lack of understanding of the molecular mechanism by which integrin β_1_ regulates csGRP78/α2M* signaling under high glucose conditions, use of only male mice in our studies, and due to stability concerns, administering the inhibitory peptide for a shorter duration in diabetic mouse models than used for antibodies.

In conclusion, in this study, we demonstrate that inhibition of csGRP78/α2M* interaction significantly reduces the production of pro-fibrotic matrix proteins by several kidney cells and reduces fibrosis in diabetic and nondiabetic CKD models. These data support targeting this ligand/receptor interaction as a therapeutic approach for inhibition of kidney fibrosis. Antibody therapeutics are clinically well established, with peptide therapeutics gaining traction. Both are well suited for inhibition of protein-protein interactions in the extracellular space. Indeed, there are currently over 200 peptides in clinical trials or preclinical development, with glucagon-like peptide 1 receptor agonists now in widespread use for several indications (47). Future studies will focus on further development and efficacy testing of csGRP78/α2M* inhibitors, as well as defining the utility of assessing urine α2M* as a potential biomarker of disease progression.

## Methods

### Sex as a biological variable.

Our studies examined male mice. Female Akita mice do not develop a significant elevation in glucose or kidney fibrosis (50).

### Cell culture.

Primary rat renal fibroblasts (Cell Biologics, RN-6016) and immortalized human PTEC (HK2 cells, ATCC) were cultured in Dulbecco’s modified Eagle medium (DMEM)/F12 supplemented with 10% fetal bovine serum (FBS). Primary human PTEC (Lonza, CC-1553, 18TL1.19985) were cultured in 10% FBS in DMEM/F12. Immortalized prostate cancer cells (1LN) were cultured in RPMI 1640 medium supplemented with 10% FBS. All cells were supplemented with streptomycin (100 μg/mL) and penicillin (100 μg/mL) and stored/grown at 37°C in 95% O_2_ and 5% CO_2_. The day prior to treatment, PTEC were serum-deprived in medium with 0.5% FBS and renal fibroblasts and mesangial cells with 1% BSA. Cells were treated with high glucose (30 mM, Sigma, G7201) or TGF-β1 (5 ng/mL, R&D Systems) with or without the following: csGRP78/α2M* inhibiting peptide (previously shown to block high glucose responses in mesangial cells, CLIGRTWNDPSVQQDIKFL) (4) or control scrambled peptide (GTNKSQDLWIPQLRDVFI) (both at 100 nM, generated in-house); csGRP78 neutralizing antibody, which blocks csGRP78/α2M* interaction (C38, 10 μg, generated in-house) (4, 14); α2M* neutralizing mouse monoclonal antibody, clone 16-11-17, which binds specifically to the α2M receptor binding domain (Fα2M, previously described in ref. 48; 10 μg, generated in-house); integrin β_1_ neutralizing antibody (10 μg, BioLegend, 303036); TGF-β1 neutralizing antibody (10 μg, R&D Systems, MAB1835); or preadsorbed IgG control (10 μg, R&D Systems, MAB002).

### Protein extraction and Western blotting.

Cells were lysed with cell lysis buffer containing protease and phosphatase inhibitors as previously described (49). Cellular debris was separated from cell lysate by centrifugation at 16,800 *g* rpm for 10 minutes at 4°C. Equal concentrations of proteins were separated using SDS-PAGE and immunoblotted with the following antibodies: GRP78 (1:1,000, BD Biosciences, 610979), Na/K ATPase (1:1,000, Novus, NB300-146), α2M (1:1,000, Invitrogen, MA5-38211), Fα2M (1:1,000, generated in-house) (48), collagen IV (Col IV) (1:1,000, Novus, NB120-6586), fibronectin (FN) (1:1,000, BD Biosciences, 610078), pSmad3 Ser473/475 (1:4,000, Novus, NBP1-77836), total Smad3 (1:1,000, Abcam, ab-40854), YAP (1:1,000, New England Biolabs [NEB], 14074T), TAZ (1:1,000, BD Biosciences, 560235), pAkt S473 (1:1,000, Cell Signaling Technology, 9271), total Akt (1:1,000, Cell Signaling Technology, 9272), pFAK Tyr397 (1:1,000, Cell Signaling Technology, 3283), total FAK (1:1,000, Santa Cruz Biotechnology, sc-558), and α-tubulin (1:5,000, Sigma, T6074).

Conditioned media were run on a nondenaturing polyacrylamide gel. Membranes were probed for the conformationally changed and more rapidly migrating α2M*. Proteins in the media could not be normalized, but each experimental well was plated to the same confluence with no difference observed at the time of media collection. Equal volumes of media were run. Nativemark unstained protein ladder (Thermo Fisher Scientific) confirmed band location.

### Biotinylation.

For surface protein extraction, cells were washed 3 times with cold phosphate-buffered saline (PBS) with 2.5 mM CaCl_2_ and 1 mM MgCl_2_ and incubated with EZ-linked Sulfo-Biotin for 30 minutes (Pierce, 21331). Cells were then washed with quenching buffer containing 0.1 M glycine in PBS to remove excess Sulfo-Biotin. After lysis, samples were clarified and incubated overnight in a 50% Neutravidin slurry with agitation (Thermo Fisher Scientific, P129200). The following day, beads were washed 5 times with lysis buffer and cleaved from the cell surface proteins by boiling at 100°C for 10 minutes in 2× protein sample buffer. Proteins were analyzed using SDS-PAGE and immunoblotting.

### Intracellular calcium assay.

1LN cells were loaded with the calcium indicator Fura-2AM (5 μM, Abcam, ab-120873) in HBSS for 45 minutes at 37°C in the dark. Baseline fluorescence readings of 1LN cells in HBSS were taken every minute for 5 minutes using a temperature-controlled fluorescent microplate reader (Gemini EM Spectra Max, Molecular Devices) set to 340 and 380 nm excitation and 510 nm emission. HBSS was replaced with conditioned media (DMEM with no phenol red) from HK2, human PTEC, or renal fibroblast cells, and then readings were taken every minute for 15 minutes. Intracellular calcium concentrations were determined by calculating the ratio of fluorescence signal (340/380 nm).

### Luciferase assays.

Cells were transfected at 50% confluence with the Smad3-responsive reporter construct, CAGA_12_-luciferase (provided by M. Bilandzic, Prince Henry’s Institute, Melbourne, Victoria, Australia), or with TEAD-luciferase (Addgene plasmid #34615) to assess YAP/TAZ activity for 12 hours (HK2 cells) or 18 hours (renal fibroblasts and mesangial cells). Cells were starved and treated as described above. At time of harvest, cells were lysed using Reporter Lysis Buffer (Promega) and frozen overnight at –80°C. Lysates were scraped and centrifuged at 16,800 *g*, and luciferase activity was measured using the Luciferase Assay System (Promega) and a SpectraMax L Microplate Reader (Molecular Devices) set to measure luminescence. Samples were normalized using the β-Galactosidase Enzyme Assay System (Promega) with a plate reader set to 420 nm absorbance (SpectraMax Plus 384 Microplate Reader, Molecular Devices).

### Experimental animals.

Kidneys were harvested from type 1 diabetic Akita mice (C57BL/6-Ins2^Akita^/J, The Jackson Laboratory) at 40 weeks of age. To generate a type 1 diabetic model that developed tubulointerstitial fibrosis earlier than 40 weeks of age, we used Akita mice expressing hypermorphic alleles for TGF-β1 (HH), resulting in overexpression of TGF-β1, as previously described (22). HH mice lacking the insulin 2 gene mutation were used as controls. Mice were bred for the study, with diabetes diagnosed by a positive urine glucose test on dipstick (Bayer Multistix) at 6 weeks of age. At 16 weeks of age, tail vein blood glucose was measured using a glucometer, and mice with values > 17 mM were enrolled as diabetics into the study. Three separate studies were conducted: (a) treatment with C38 (made in-house) or the isotype control IgG2b (BioXcell, BE0086) antibodies; (b) Fα2M (made in-house) or the isotype control IgG1 (BioXcell, BE0083) antibodies; or (c) P19 or scrambled control peptide (both made in-house). In both antibody studies, mice were treated with 5 mg/kg IP twice a week for 12 weeks. In the peptide study, mice were implanted with a 2-week osmotic minipump (Alzet, 1002) delivering 35 μg/d at 22 weeks of age. The minipump was replaced at 2 weeks, for a total of 4 weeks of treatment. Diabetic mice that developed ketonuria (dipstick, Bayer Multistix) or had progressive weight loss were administered ¼ of an insulin pellet (LinShin Canada) to maintain body weight while maintaining hyperglycemia. The week prior to sacrifice, urine was collected for measurement of ACR (Albuwell, Exocell-albumin; Crystal Chem-creatinine). GFR was measured just prior to sacrifice by measuring the clearance of FITC–labeled sinistrin (Fresenius Kabi Linz). After 12 weeks of treatment for antibodies or 4 weeks for peptide, mice were anesthetized, then perfused with saline, and kidneys were harvested for further analysis.

To generate the UUO model of fibrosis, the left ureter was ligated close to the renal pelvis in 8-week-old male C57BL/6 mice (Charles River Laboratories). Sham mice were anesthetized, and their kidneys manipulated without ligation. Four separate studies were conducted: (a) a time course, with harvest at 1, 7, 14, and 21 days after UUO or sham operation to assess target protein expression; (b) a treatment study, with Fα2M or control IgG1 antibody administered by IP injection (5 mg/kg, twice a week) starting the day after UUO surgery, with harvest at 10 days after UUO; (c) a treatment study, in which a 7-day osmotic minipump (Alzet, 1007D) was implanted 3 days after UUO surgeries to deliver functional or scrambled csGRP78/α2M* inhibitory peptide at 30 or 150 μg/d until harvest at 10 days; and (d) a treatment study, in which a 7-day osmotic minipump (Alzet, 1007D) was implanted 7 days after UUO surgeries to deliver functional or scrambled peptide at 150 μg/d until harvest at 14 days.

### Immunofluorescence.

OCT-preserved kidney sections (10 μm) were fixed (3.7% paraformaldehyde) and permeabilized (0.2% Triton X-100). These steps were omitted for csGRP78 assessment to minimize staining of intracellular GRP78. Tissues were stained for GRP78 (Abcam, ab21685, 1:50,000), α2M* (Fα2M, 1:100), and/or megalin (Biocell Scientific, 1:200). Secondary antibodies used were anti-rabbit (AF488, Invitrogen, A21206) and anti-mouse (AF488, Invitrogen, A21202). Images were captured using the Olympus BX41 microscope at 20× original magnification. The ImageJ colocalization plugin was used to create a colocalization mask of areas expressing both csGRP78 and WGA (1:400, AF594, Invitrogen, W11262), which allowed localization of GRP78 to the cell surface or to create a colocalization mask for areas of the kidney cortex coexpressing csGRP78 and megalin or α2M* and megalin. Quantification was completed using ImageJ (NIH). Scale bar in images indicates 20 μm.

### Immunohistochemistry.

For immunohistochemistry, 4 μm paraffin-embedded kidney sections were deparaffinized and then stained with PSR (Polysciences Inc., 24901-250) or with the following: FN (1:500, proteinase K 40 μg/mL for 5 minutes, Sigma, F3648), pFAK Tyr397 (1:2,000, proteinase K 40 μg/mL for 5 minutes, GeneTex, GTX129840), pSmad3 (1:500, citric acid steam 30 minutes, Novus, NBP1-77836), YAP (1:200, citric acid steam 30 minutes, NEB, 14074), α-SMA (1:1,000, citric acid steam 30 minutes, Thermo Fisher Scientific, MA1-06110), pAkt (1:50, citric acid steam 30 minutes, NEB, 4060), CD3 (1:750, citric acid steam 30 minutes, Serotec, MCA1477), and F4/80 (staining completed by the McMaster Immunology Research Centre, CORE Histology Research Services). Periodic acid–Schiff staining was completed to assess glomerular hypertrophy by measurement of glomerular cross-sectional area as described previously (51). Images were captured at 20× magnification (40× for glomerular volume) and quantified using ImageJ software. Scale bar in images indicates 20 μm (and 10 μm for glomerular volume images).

### Surface plasmon resonance.

Surface plasmon resonance experiments were performed on a T200 BIAcore instrument (Cytiva). α2M* was immobilized on a CM5 chip using amine chemistry (Cytiva) to 9600 resonance units (RU). Flow cell 1 was activated similarly and blocked with 1 M ethanolamine. Dilutions of Fα2M were made in 20 mM HEPES, 150 mM NaCl, pH 7.4, containing 0.05% Tween 20. Samples were injected into the flow cells at 30 μL/min for 30 seconds, and dissociation was monitored for 200 seconds. Flow cells were regenerated with 10 mM glycine, pH 2.5. RU values were corrected for the blank flow cell and for 0 nM Fα2M. Maximal RU values were plotted against Fα2M concentration and evaluated by steady-state analysis using BIAevaluation software (Cytiva).

### Statistics.

Analysis was completed using GraphPad Prism (version 10.2). Data points were assessed for outliers within each group using the Grubbs outlier test, and outliers were removed. Data points within groups were assessed for normality using the Shapiro-Wilk normality test. If this was passed, data were further analyzed using either a 2-tailed *t* test for comparison between 2 groups or a 1-way ANOVA, with Tukey’s post hoc test used for comparison between 3 or more groups. However, if a group failed the normality test, data were analyzed using the Mann-Whitney *U* test for comparison between 2 groups or Kruskal-Wallis, with Dunn’s post hoc test for comparison between 3 or more groups. Statistical significance was set at *P* ≤ 0.05, and data are presented as mean ± SEM.

### Study approval.

All studies were conducted in accordance with McMaster University, the Canadian Council on Animal Care, and Animal Research: Reporting of In Vivo Experiments guidelines. Animal studies were approved by McMaster Animal Research Ethics Board.

### Data availability.

The data obtained and presented in this article are reported in the Supporting Data Values file.

## Author contributions

JT, KP, RL, MAM, and UFB performed experiments. ZY, SK, and AFR provided peptide design and synthesis expertise and synthesized peptides. NU and MBL performed RNA-sequencing analysis. IKN, MM, CL, and BG supported animal studies. MK provided HH mice. YP created and provided the Fα2M antibody and provided intellectual input. SVP created and provided the C38 antibody and provided intellectual input. JCF and JIW conducted surface plasmon resonance experiments and provided intellectual input. JT and JCK conceived the experimental design. JT analyzed the data. JT and JCK wrote and edited the manuscript. All authors read and accepted the final manuscript.

## Funding support

- Canadian Institutes of Health Research (JCK, PJT-148628), Diabetes Canada (JCK, OG-3-23-5791-JK), and Lundbeck Foundation (KP, R347-2020-2388).
- Ontario Graduate Scholarship award and the 2021 RSJH Studentship award (JT).
- Canada Research Chair (Tier I) in Thrombosis and the Heart and Stroke Foundation J. F. Mustard Chair in Cardiovascular Research (JIW).

## Supplementary Material

Supplemental data

Unedited blot and gel images

Supporting data values

## Version 1 12/22/2025

Electronic publication

## References

[1] Kovesdy CP (2022). Epidemiology of chronic kidney disease: an update 2022. Kidney Int Suppl (2011).

[2] Kalantar-Zadeh K (2021). Chronic kidney disease. Lancet.

[3] Van Krieken R (2019). Cell surface expression of 78-kDa glucose-regulated protein (GRP78) mediates diabetic nephropathy. J Biol Chem.

[4] Trink J (2021). Activated alpha 2-macroglobulin is a novel mediator of mesangial cell profibrotic signaling in diabetic kidney disease. Biomedicines.

[5] Trink J (2022). Integrin β1/cell surface GRP78 Complex regulates TGFβ1 and its profibrotic effects in response to high glucose. Biomedicines.

[6] Trink J (2023). Cell surface GRP78 regulates TGFβ1-mediated profibrotic responses via TSP1 in diabetic kidney disease. Front Pharmacol.

[7] Voelker J (2017). Anti-TGF-b1 antibody therapy in patients with diabetic nephropathy. J Am Soc Nephrol.

[8] Zhang Y (2021). Signaling pathways involved in diabetic renal fibrosis. Front Cell Dev Biol.

[9] Chang J (2021). Update on the mechanisms of tubular cell injury in diabetic kidney disease. Front Med (Lausanne).

[10] Yuan Q (2022). Signaling pathways of chronic kidney diseases, implications for therapeutics. Signal Transduct Target Ther.

[11] Chang K-J (1975). Membrane receptors as general markers for plasma membrane isolation procedures. The use of 125-I-labeled wheat germ agglutinin, insulin, and cholera toxin. J Biol Chem.

[12] Misra UK (2005). The role of MTJ-1 in cell surface translocation of GRP78, a receptor for alpha 2-macroglobulin-dependent signaling. J Immunol.

[13] Gopal U (2016). Activated α2-macroglobulin regulates transcriptional activation of c-MYC target genes through cell surface GRP78 protein. J Biol Chem.

[14] De Ridder GG (2012). A murine monoclonal antibody directed against the carboxyl-terminal domain of GRP78 suppresses melanoma growth in mice. Melanoma Res.

[15] Meng XM (2015). TGF-β/Smad signaling in renal fibrosis. Front Physiol.

[16] Gu YY (2020). Diverse role of TGF-β in kidney disease. Front Cell Dev Biol.

[17] Anorga S (2018). Deregulation of Hippo-TAZ pathway during renal injury confers a fibrotic maladaptive phenotype. FASEB J.

[18] Patel S (2019). Rac-GTPase promotes fibrotic TGF-β1 signaling and chronic kidney disease via EGFR, p53, and Hippo/YAP/TAZ pathways. FASEB J.

[19] Seunghyeok Choi (2022). Hyperactivation of YAP/TAZ drives alterations in mesangial cells through stabilization of N-Myc in diabetic nephropathy. J Am Soc Nephrol.

[20] Szeto SG (2016). YAP/TAZ are mechanoregulators of TGF-β-Smad signaling and renal fibrogenesis. J Am Soc Nephrol.

[21] Gopal U (2019). Targeting cell surface GRP78 enhances pancreatic cancer radiosensitivity through YAP/TAZ protein signaling. J Biol Chem.

[22] Hathaway CK (2015). Low TGFβ1 expression prevents and high expression exacerbates diabetic nephropathy in mice. Proc Natl Acad Sci U S A.

[23] Martínez-Klimova E (2019). Unilateral ureteral obstruction as a model to investigate fibrosis-attenuating treatments. Biomolecules.

[24] Huang R (2023). Kidney fibrosis: from mechanisms to therapeutic medicines. Signal Transduct Target Ther.

[25] Tang PCT (2021). TGF-β1 signaling: immune dynamics of chronic kidney diseases. Front Med (Lausanne).

[26] Sureshbabu A (2016). TGF-β signaling in the kidney: profibrotic and protective effects. Am J Physiol Renal Physiol.

[27] Liang M (2017). Yap/Taz deletion in Gli^+^ cell-derived myofibroblasts attenuates fibrosis. J Am Soc Nephrol.

[28] Inazaki K (2004). Smad3 deficiency attenuates renal fibrosis, inflammation,and apoptosis after unilateral ureteral obstruction. Kidney Int.

[29] Ma LJ (2003). Transforming growth factor-beta-dependent and -independent pathways of induction of tubulointerstitial fibrosis in beta6(–/–) mice. Am J Pathol.

[30] Du Y (2019). PTEN improve renal fibrosis in vitro and in vivo through inhibiting FAK/AKT signaling pathway. J Cell Biochem.

[31] Speer T (2022). Targeting innate immunity-driven inflammation in CKD and cardiovascular disease. Nat Rev Nephrol.

[32] Rehman AA (2013). α-2-Macroglobulin: a physiological guardian. J Cell Physiol.

[33] Cater JH (2019). Alpha-2-macroglobulin, a hypochlorite-regulated chaperone and immune system modulator. Oxid Med Cell Longev.

[34] Fan G (2021). Urine proteomics identifies biomarkers for diabetic kidney disease at different stages. Clin Proteomics.

[35] Menon R (2020). Single cell transcriptomics identifies focal segmental glomerulosclerosis remission endothelial biomarker. JCI Insight.

[36] Hills CE, Squires PE (2011). The role of TGF-β and epithelial-to mesenchymal transition in diabetic nephropathy. Cytokine Growth Factor Rev.

[37] Gewin LS (2018). Renal fibrosis: primacy of the proximal tubule. Matrix Biol.

[38] Klinkhammer BM (2017). Treatment of renal fibrosis-turning challenges into opportunities. Adv Chronic Kidney Dis.

[39] Gava AL (2012). Effects of 5/6 nephrectomy on renal function and blood pressure in mice. Int J Physiol Pathophysiol Pharmacol.

[40] Wang L (2021). TGF-beta as a master regulator of diabetic nephropathy. Int J Mol Sci.

[41] Miranda MZ (2017). TGF-β1 regulates the expression and transcriptional activity of TAZ protein via a Smad3-independent, myocardin-related transcription factor-mediated mechanism. J Biol Chem.

[42] Szeto SG (2016). YAP/TAZ are mechanoregulators of TGF-*β*-Smad signaling and renal fibrogenesis. J Am Soc Nephrol.

[43] Wong JS (2016). Hippo signaling in the kidney: the good and the bad. Am J Physiol Renal Physiol.

[44] Wang S (2016). TGF-β/Smad3 signalling regulates the transition of bone marrow-derived macrophages into myofibroblasts during tissue fibrosis. Oncotarget.

[45] Nikolic-Paterson DJ (2014). Macrophages promote renal fibrosis through direct and indirect mechanisms. Kidney Int Suppl (2011).

[46] Vandooren J, Itoh Y (2021). Alpha-2-macroglobulin in inflammation, immunity and infections. Front Immunol.

[47] Wang L (2022). Therapeutic peptides: current applications and future directions. Signal Transduct Target Ther.

[48] Gurley SB (2006). Impact of genetic background on nephropathy in diabetic mice. Am J Physiol Renal Physiol.

[49] Biltoft D (2017). Fast form alpha-2-macroglobulin - a marker for protease activation in plasma exposed to artificial surfaces. Clin Biochem.

[50] Krepinsky JC (2003). Nitric oxide inhibits stretch-induced MAPK activation in mesangial cells through RhoA inactivation. J Am Soc Nephrol.

[51] Van Krieken R (2018). Inhibition of SREBP with fatostatin does not attenuate early diabetic nephropathy in male mice. Endocrinology.

[52] Dupont S (2011). Role of YAP/TAZ in mechanotransduction. Nature.

