# Supplementary material for: Inhibition of cell surface GRP78 and activated α2M interaction attenuates kidney fibrosis
Source: JCI Insight. 2025 Dec 22;10(24):e183998. doi: 10.1172/jci.insight.183998 (PMC12890533; doi:10.1172/jci.insight.183998)
Supplement: Supplemental data [file jciinsight-10-183998-s053.pdf]

Supplementary Figure File

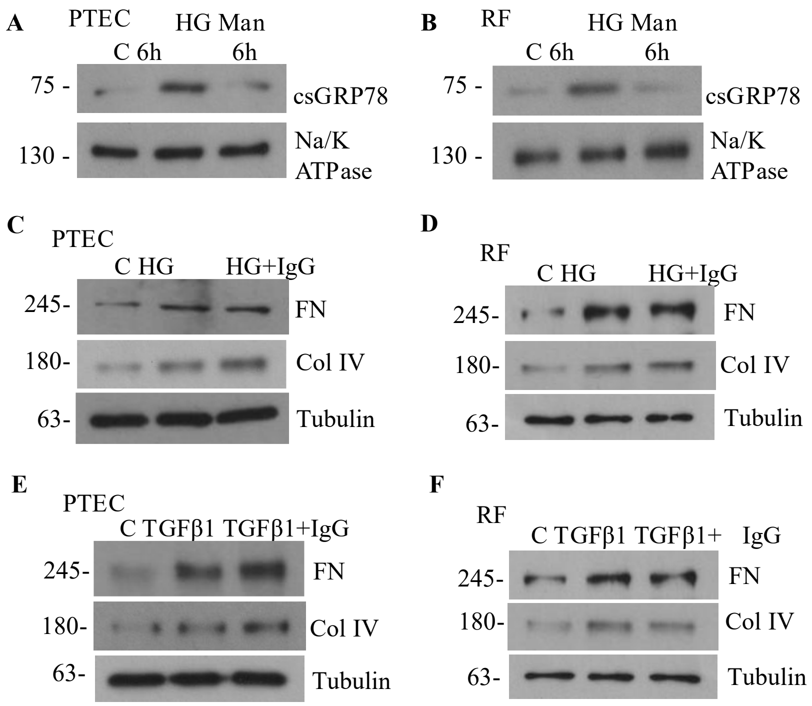

Supplementary Figure 1: The osmotic control mannitol and isotype IgG control had no biologic effect. High glucose, but not the osmotic control mannitol, induces surface translocation of GRP78 in both **(A)** PTEC and **(B)** renal fibroblasts. Increased expression of fibronectin and collagen IV by high glucose in PTEC **(C)** or renal fibroblasts **(D)** or by TGFβ1 in PTEC **(E)** or renal fibroblasts **(F)** were not affected by control IgG administration.

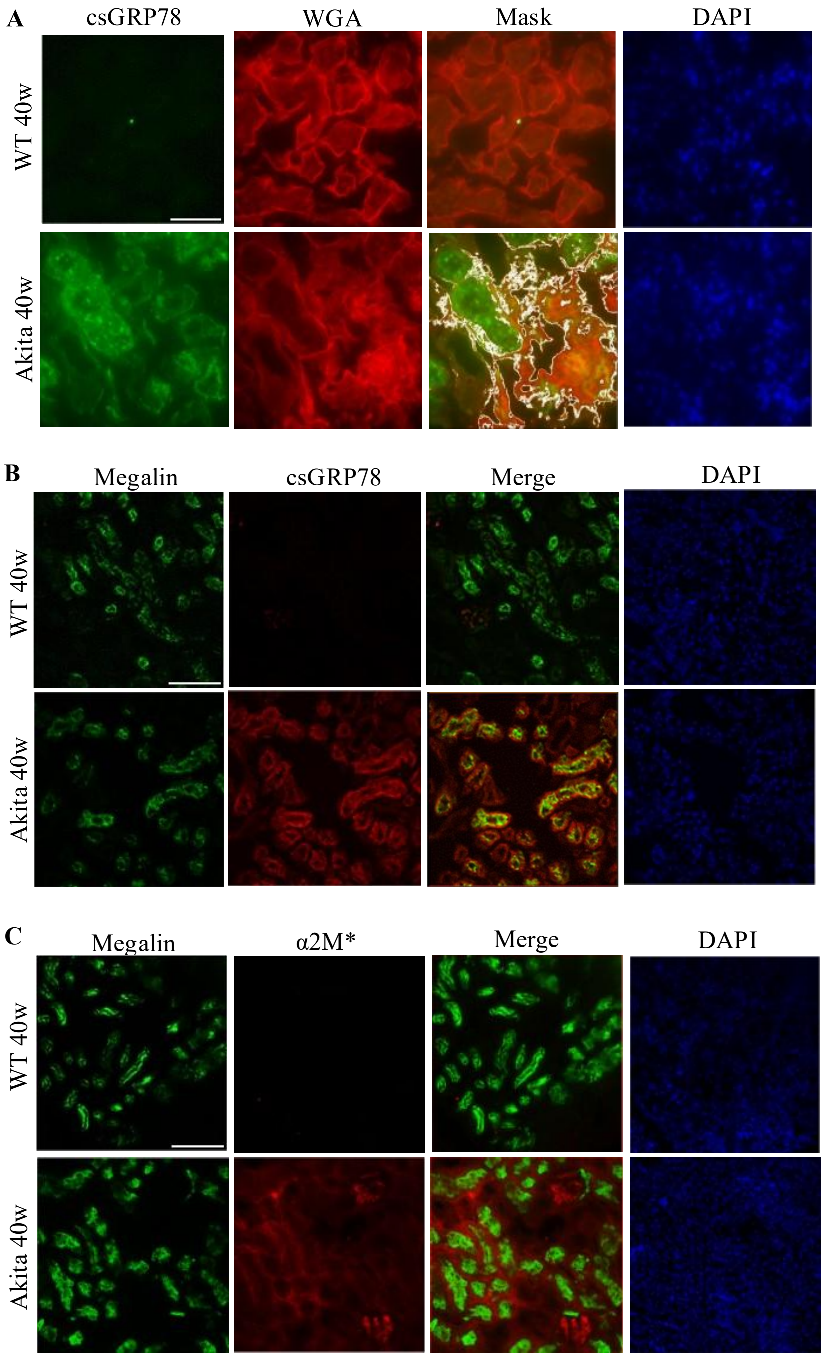

Supplementary Figure 2: Increased tubular expression of csGRP78 and α2M* was seen in kidneys of type 1 diabetic mice. (A) In 40-week-old Akita mice, increased csGRP78 expression (green) was seen by immunofluorescence of nonpermeabilized kidney sections. Cell surface location was confirmed by colocalization with the plasma membrane marker wheat germ agglutinin (WGA)(red) as seen by the white colocalization mask. Increased **(B)** csGRP78 (red) and **(C)** α2M* (green) expression were colocalized to proximal tubular cells with the PTEC marker megalin (scale bar represents 20μm in all panels except (A) where scale bar represents 10μm).

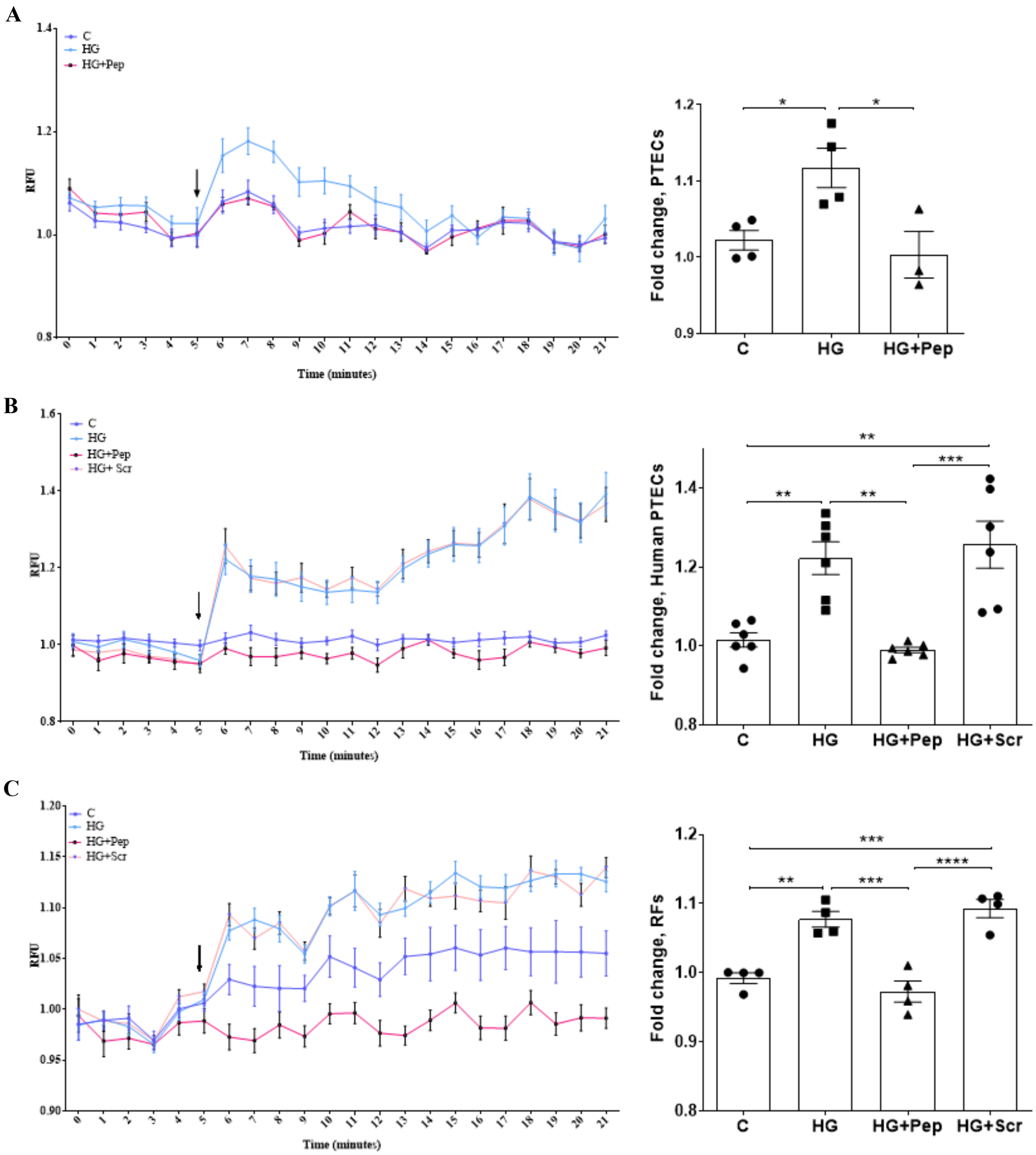

Supplementary Figure 3: Peptide inhibition prevents PTEC production of α2M*. 1LN cells which express high levels of csGPR78 were exposed to conditioned media from **(A)** HK2 cells, **(B)** primary PTEC, and **(C)** renal fibroblasts treated or not with high glucose. This induced the release of intracellular calcium stores. Inhibition of the calcium increase with the α2M* peptide inhibitor confirmed that this rise was due to csGRP78/α2M* interaction. The graph shows comparison of groups at one minute after time of treatment, which is indicated by the arrow (n = 3-6, * $p < 0.05$, ** $p < 0.01$, *** $p < 0.005$, ****$p<0.0001$).

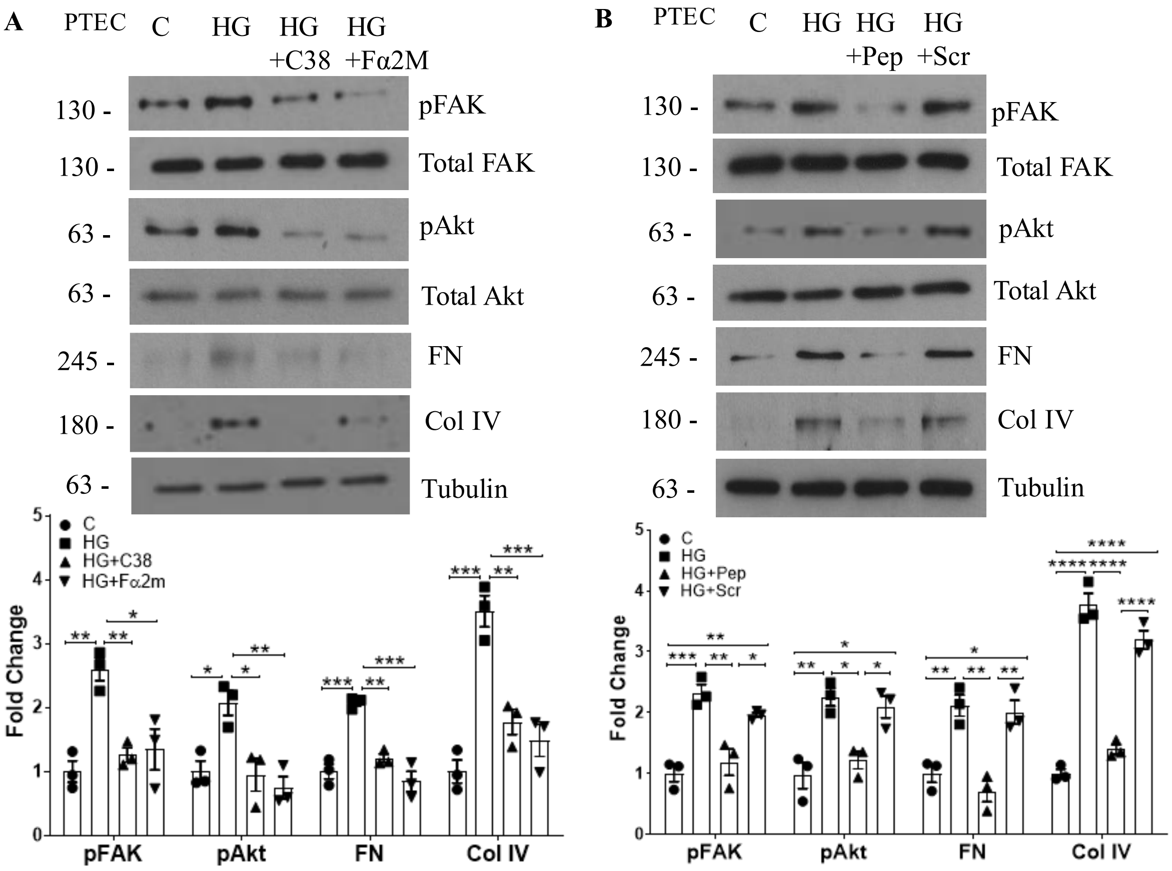

Supplementary Figure 4: csGRP78 and α2M* inhibition prevent profibrotic signaling in human PTEC. (A) High glucose (30mM, 48h)-induced activation of FAK and Akt (phosphorylation at Y397 and S473 respectively) as well as matrix production (fibronectin, FN and collagen IV, Col IV) were prevented by either csGRP78 (C38, 10µg) or α2M* (Fα2M, 10µg) inhibition in human PTEC (n= 3). **(B)** Inhibition of csGRP78/α2M* interaction with peptide (100nM), but not scrambled control peptide (100nM), prevented high glucose (30mM, 48h)-induced FAK and Akt activation as well as FN and Col IV expression in human PTEC (n= 3) **(B)** (* $p < 0.05$, ** $p < 0.01$, *** $p < 0.005$, ****$p<0.0001$).

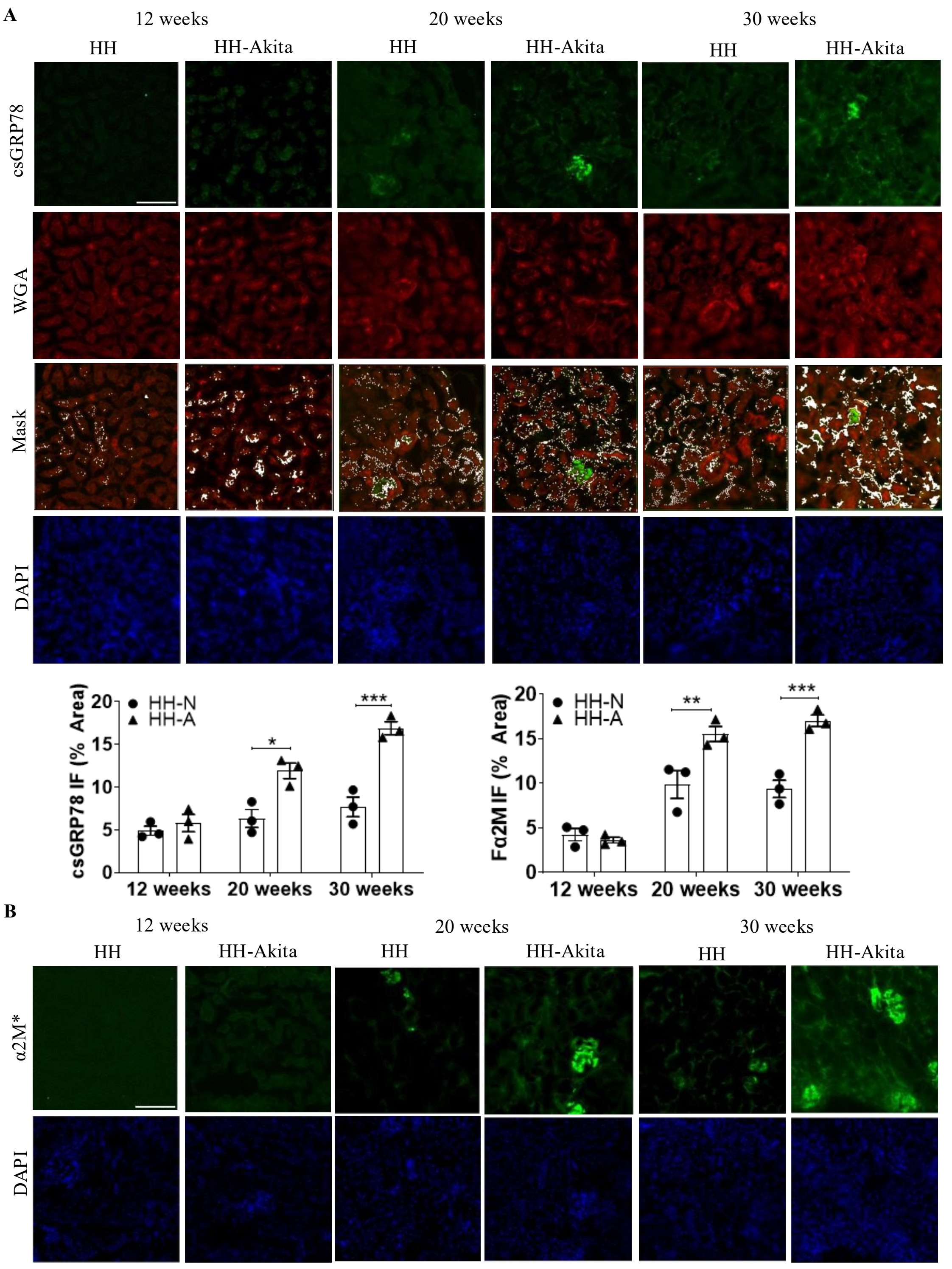

Supplementary Figure 5: Increased tubular expression of csGRP78 and α2M* was seen in kidneys of TGFβ1 overexpressing diabetic mice. (A) Expression of csGRP78 (colocalized with membrane marker WGA) and **(B)** α2M* were increased in type 1 diabetic Akita mice overexpressing TGFβ1 at 20 and 30 weeks of age, compared to non-diabetic mice overexpressing TGFβ1 mice (n= 3) (* $p < 0.05$, ** $p < 0.01$, *** $p < 0.005$, scale bar represents 20μm).

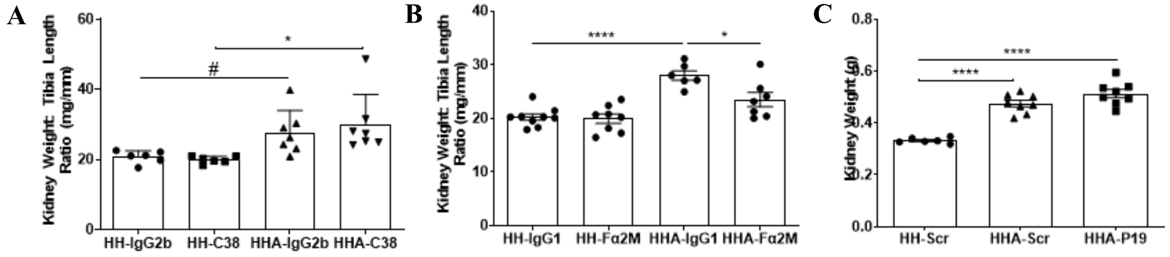

Supplementary Figure 6: α2M*, but not csGRP78 inhibition, prevented kidney hypertrophy in a type 1 diabetic mouse model. (A) Inhibition of csGRP78 with C38 did not prevent kidney hypertrophy in type 1 diabetic Akita mice overexpressing TGFβ1 (n=5-7). **(B)** Kidney hypertrophy was significantly reduced with α2M* inhibition using the Fα2M antibody (n=6-9). **(C)** Peptide inhibition of csGRP78/α2M* interaction did not prevent kidney hypertrophy in type 1 diabetic Akita mice (n= 6-8) (* $p < 0.05$, ****$p<0.0001$; significant by t-test only, # $p < 0.05$).

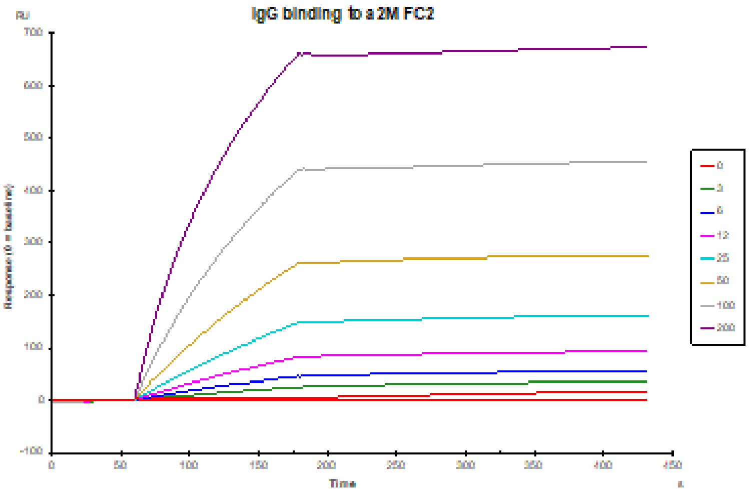

Supplementary Figure 7: Confirmation of Fα2M antibody binding to α2M*. Surface plasmon resonance shows dose-dependent binding of the Fα2M antibody to α2M*. Increasing concentrations of Fα2M (nM; inset) were injected over a flow cell containing 9600 resonance units of α2M* for 30 s and dissociation was monitored for 300 s. Sensorgram tracings reflect correction for blank flow cell 1 and the response for 0nM Fα2M.

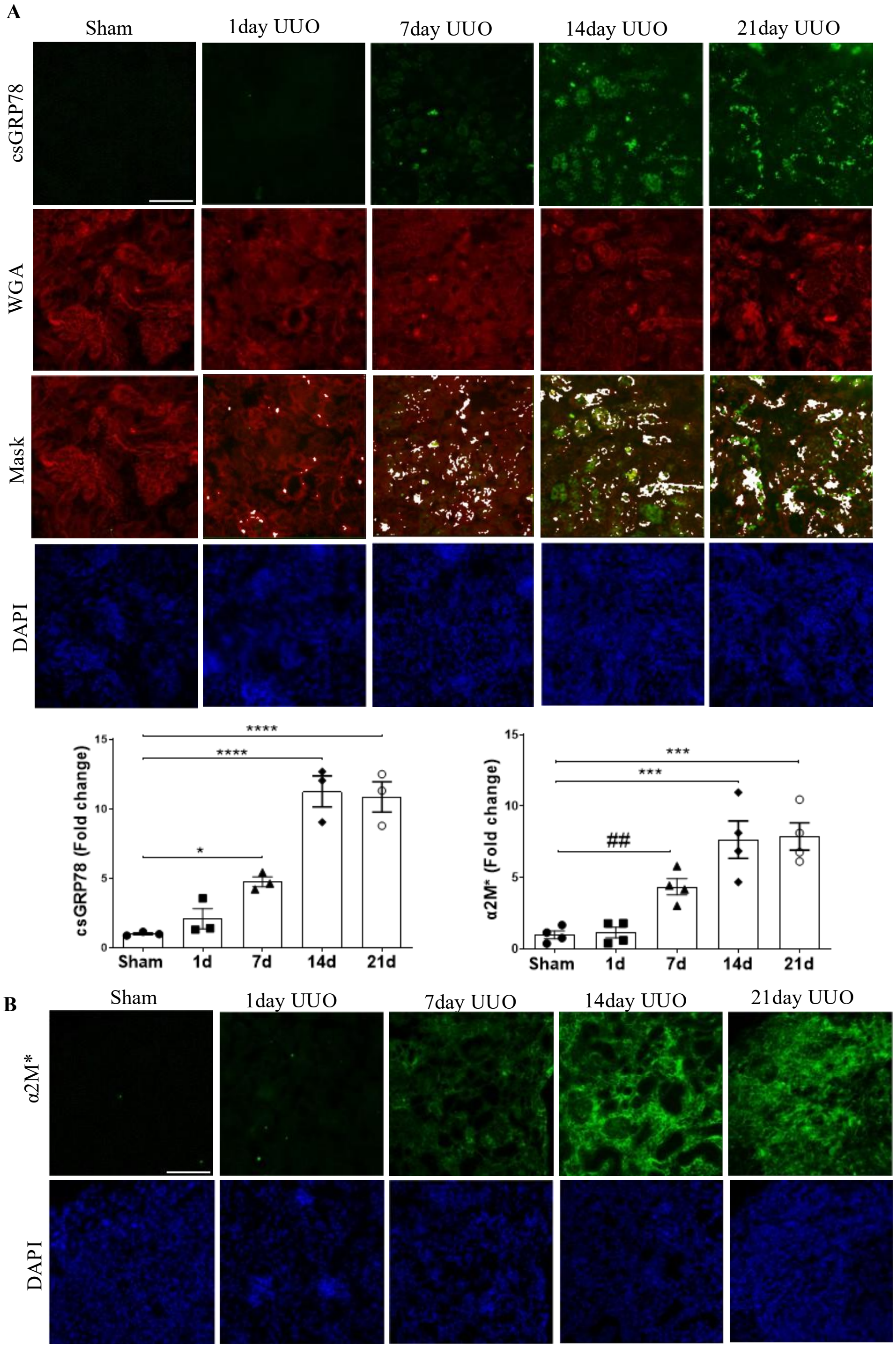

Supplementary Figure 8: Increased tubular expression of csGRP78 and α2M* was seen in kidneys after UUO. (A) Immunofluorescence staining for csGRP78 and the membrane marker WGA in nonpermeabilized sections showed increased expression in UUO mice at days 7, 14, and 21 following model creation. Sham mice notably did not express csGRP78 (n=3). **(B)** Increased α2M* expression was observed at 7 (significant by t-test only, ## $p < 0.01$), 14 and 21 days following UUO surgery. Sham operated mice did not express α2M* (n=4) (* $p < 0.05$, *** $p < 0.005$, ****$p<0.0001$, scale bar represents 20μm).

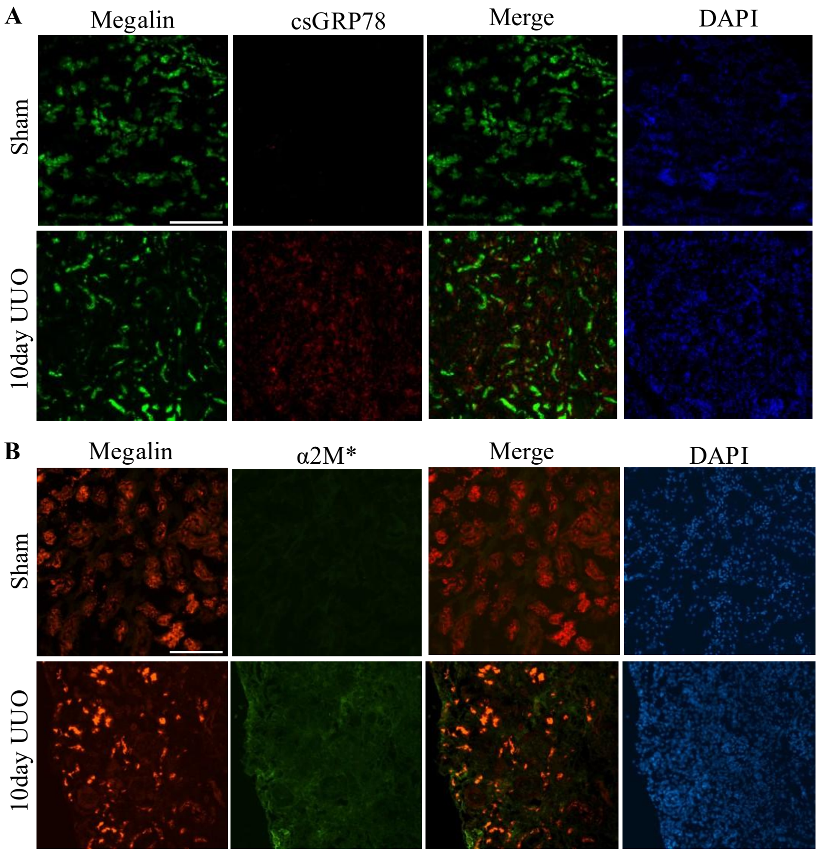

Supplementary Figure 9: Increased tubular expression of csGRP78 and α2M* was seen in the UUO model for kidney fibrosis. Increased expression and colocalization was seen of **(A)** csGRP78 and **(B)** α2M* with the PTEC marker megalin in (A) and lotus tetragonolobus lectin (LTL) in (B) in 10-day UUO mice, but not sham control mice (scale bar represents 20μm).

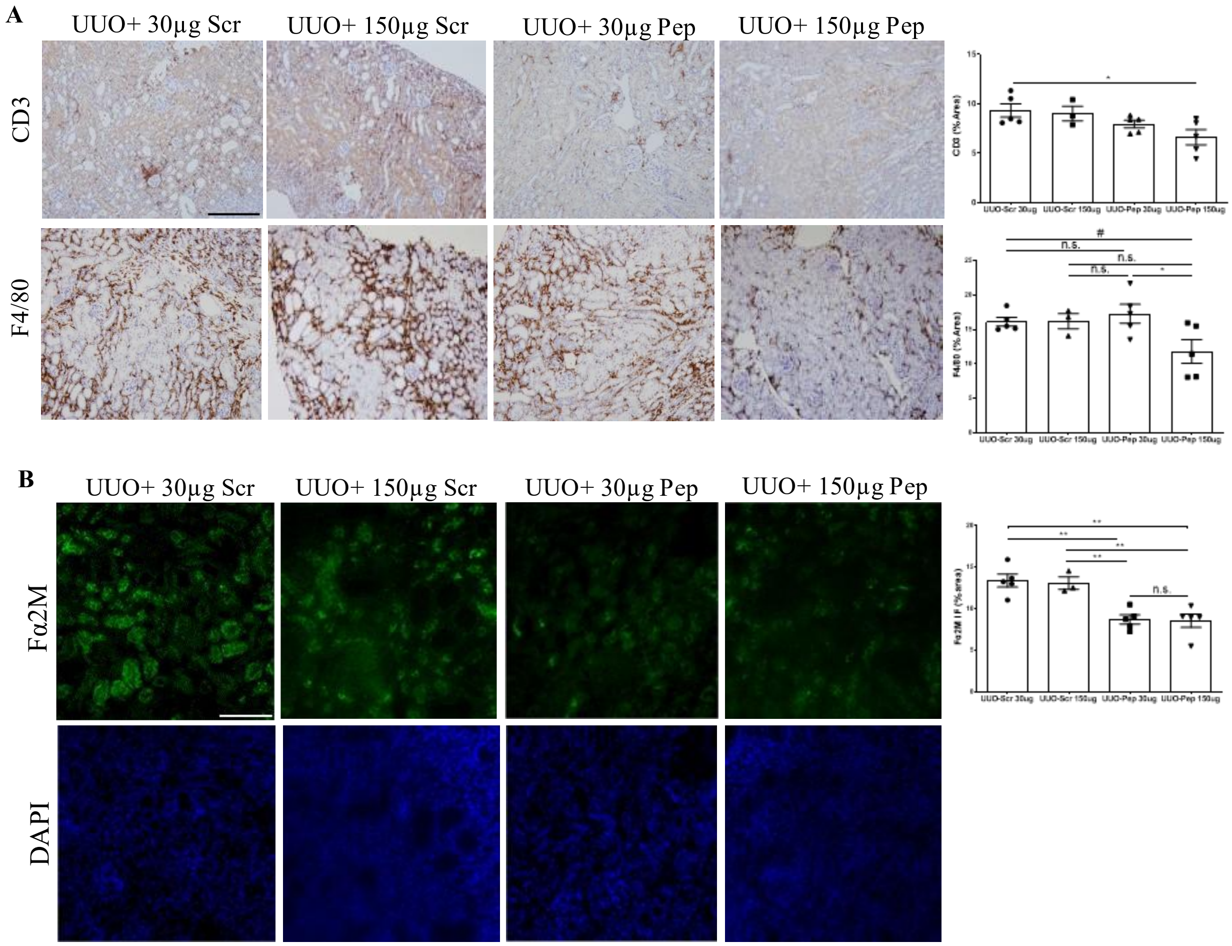

Supplementary Figure 10: Peptide inhibition of csGRP78/α2M* interaction reduces inflammatory cell infiltration and α2M* in UUO mice. (A) Infiltration of T cells (CD3) and macrophages (F4/80) was assessed by immunohistochemistry. Both were reduced by the higher dose of peptide. **(B)** α2M* was assessed by immunohistochemistry using the Fα2M antibody. Levels were reduced by both doses of peptide (n= 3-5, * $p < 0.05$, ** $p < 0.01$, *** $p < 0.005$, scale bar represents 20μm).
