# Supplementary material for: Inhibition of cell surface GRP78 and activated α2M interaction attenuates kidney fibrosis
Source: JCI Insight. 2025 Dec 22;10(24):e183998. doi: 10.1172/jci.insight.183998 (PMC12890533; doi:10.1172/jci.insight.183998)

Figure 1

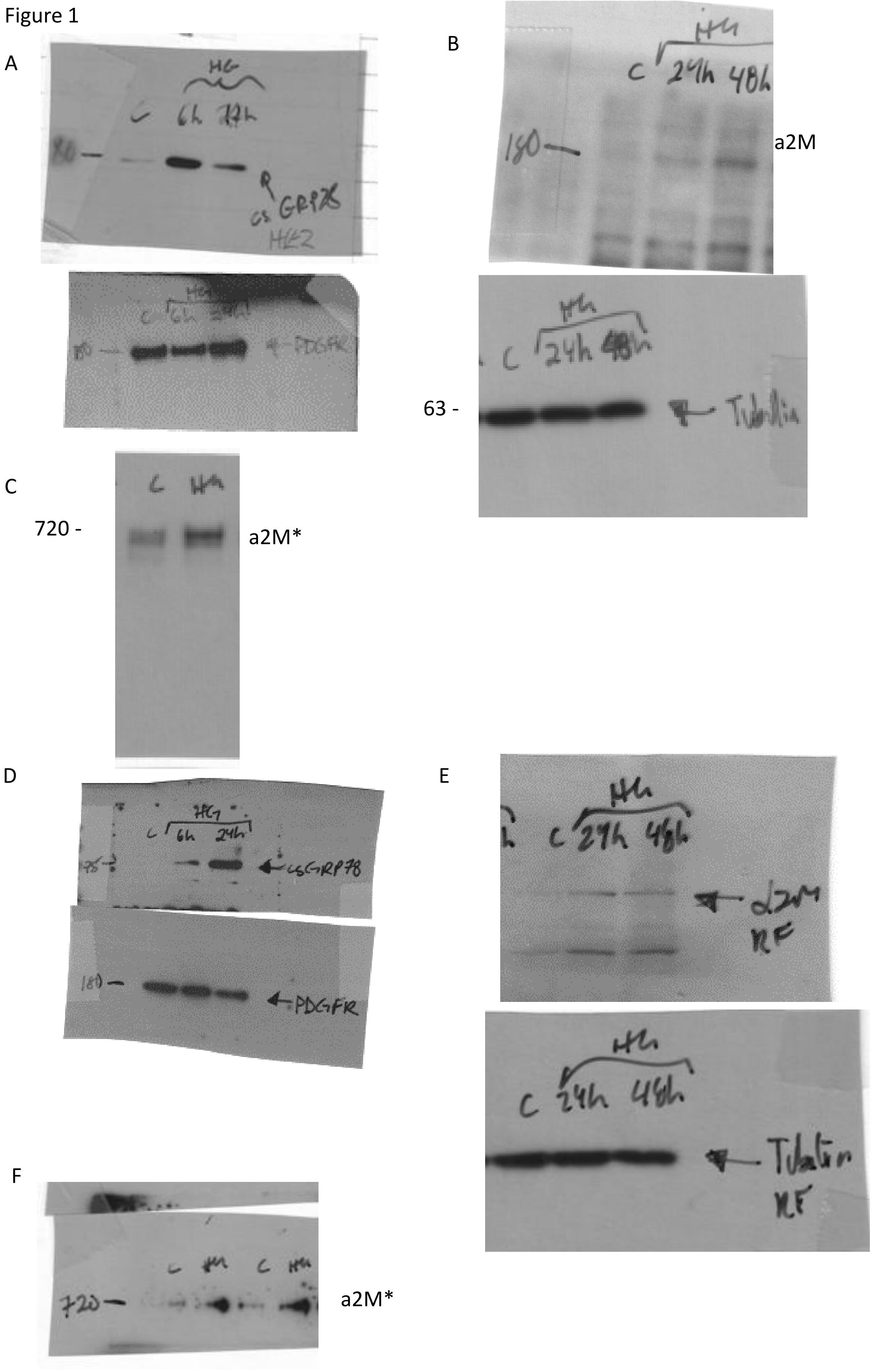

Figure 1

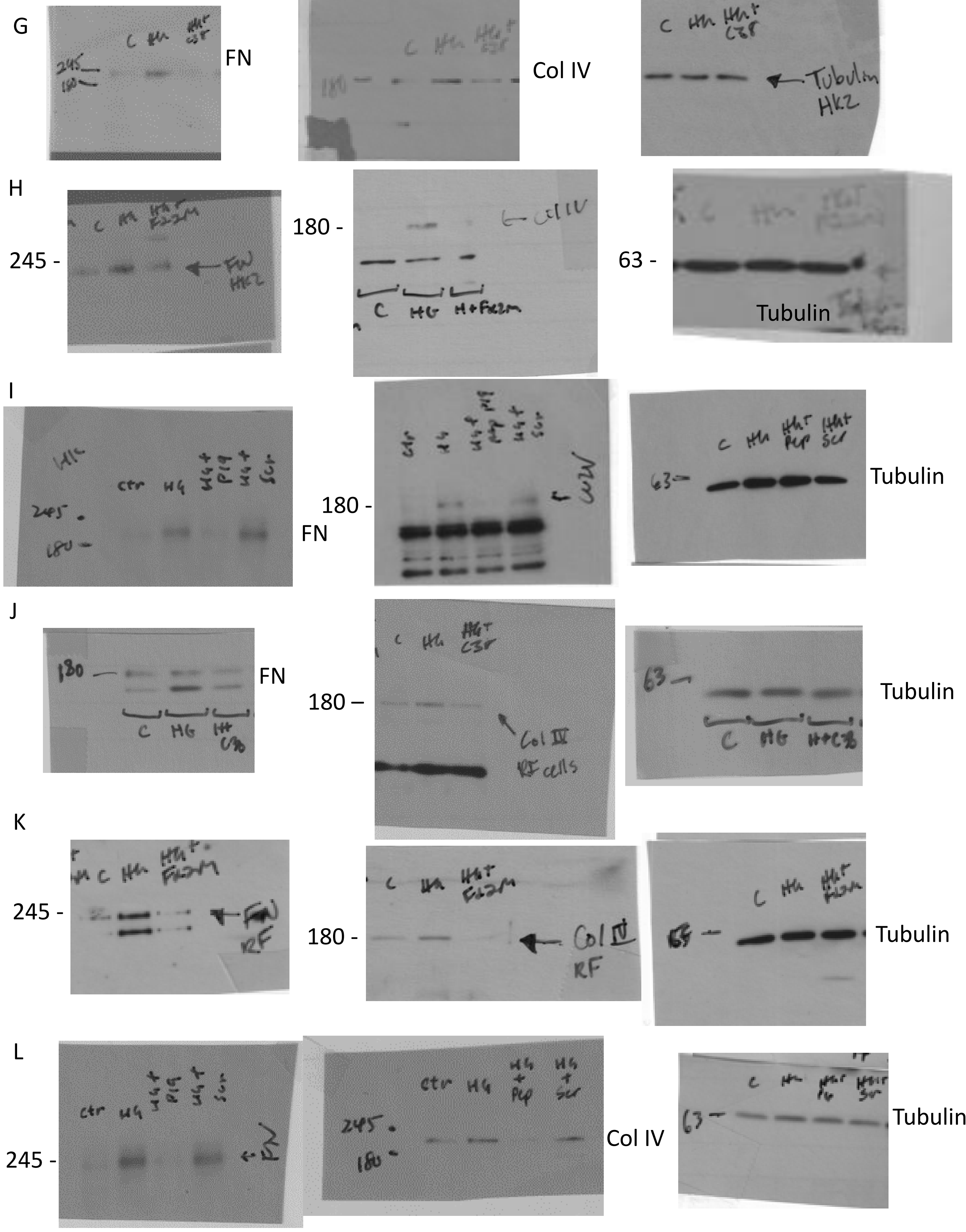
G
FN
Col IV
H
245 -
180 -
63 -
Tubulin
I
FN
180 -
Tubulin
J
FN
180 –
Tubulin
K
245 -
180 -
Tubulin
L
245 -
Col IV
Tubulin

Figure 2

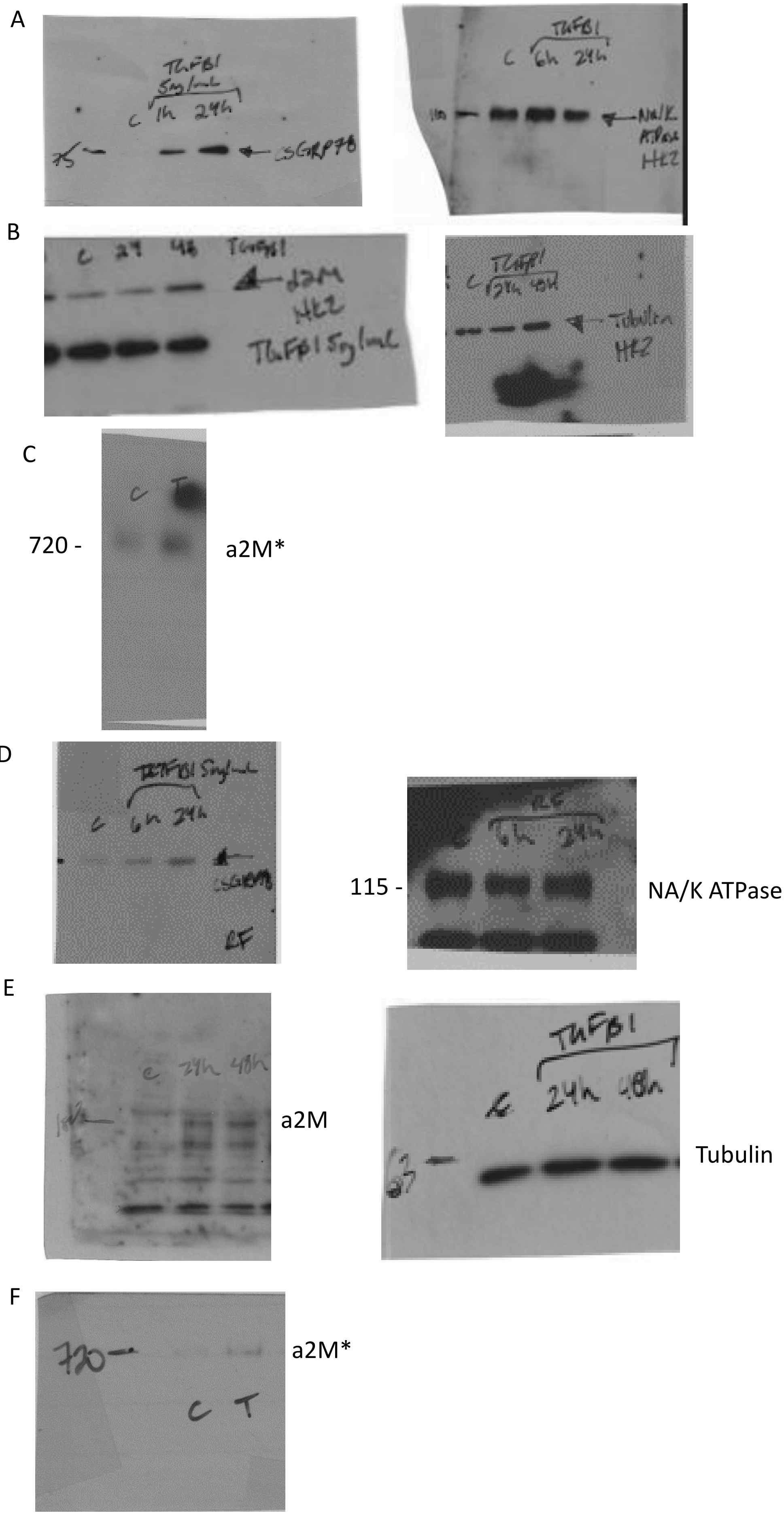

Figure 2

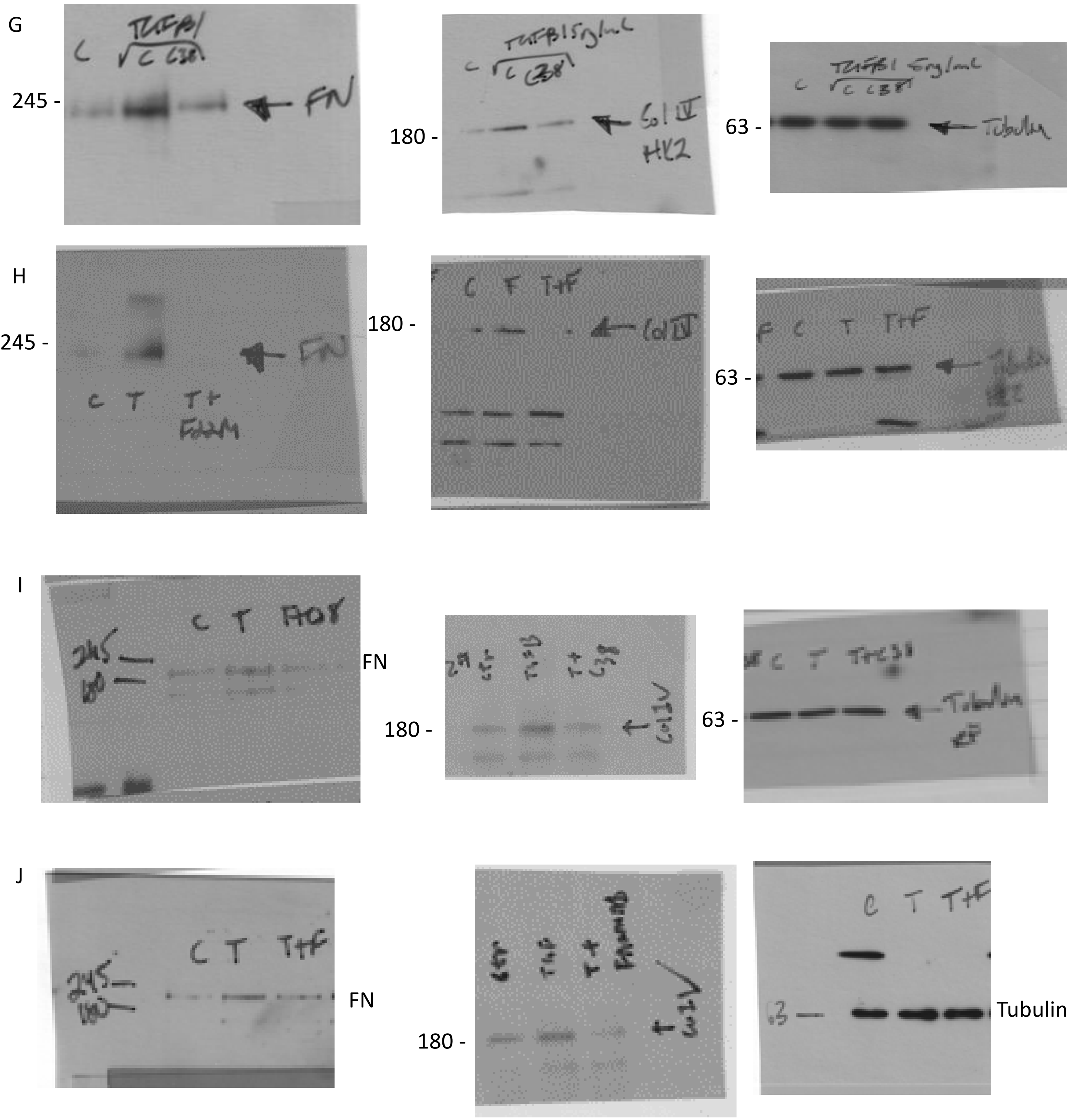

Figure 3

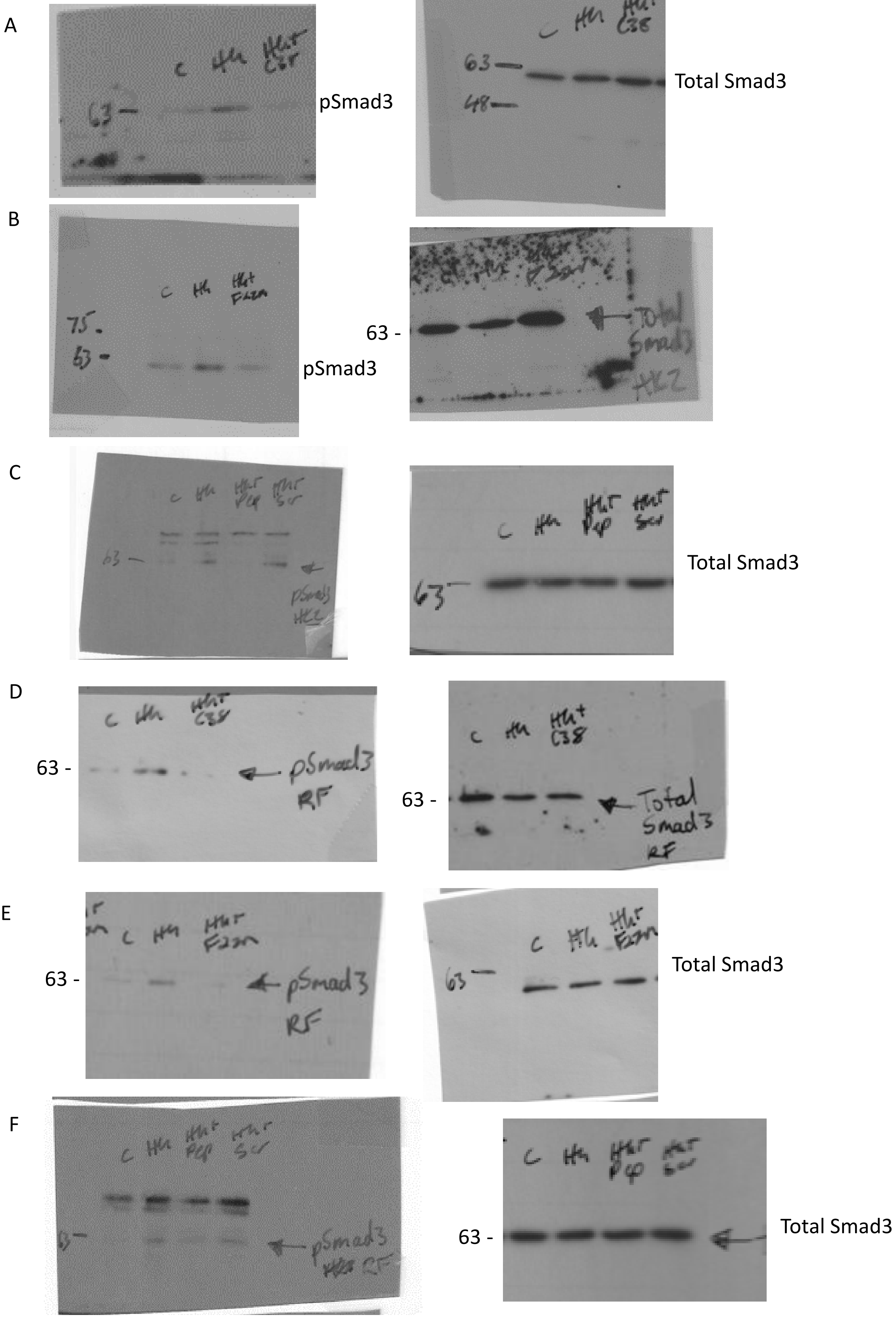
A
pSmad3
Total Smad3
B
pSmad3
63 -
C
Total Smad3
D
63 -
63 -
E
63 -
Total Smad3
F
63 -
Total Smad3

Figure 3

G

63 -

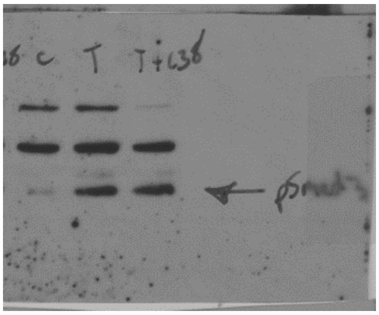

63 -

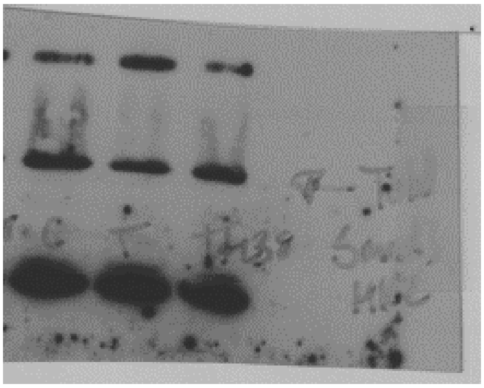

H

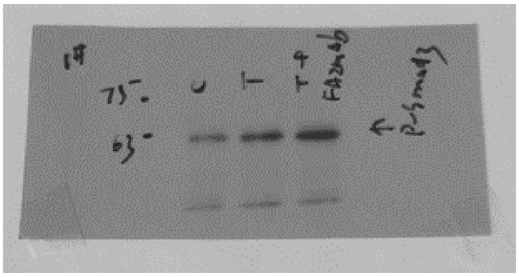

63 -

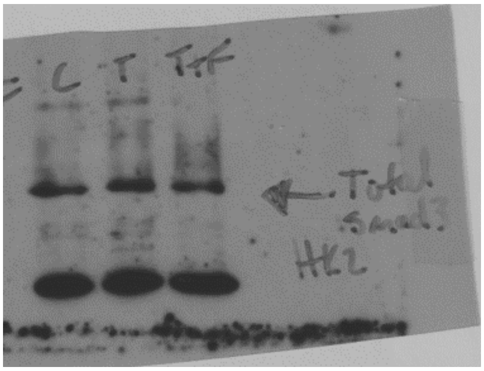

J

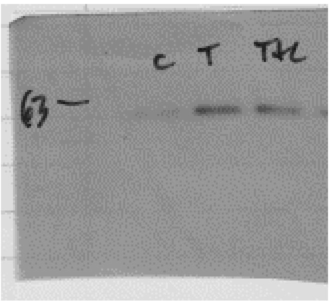

pSmad3

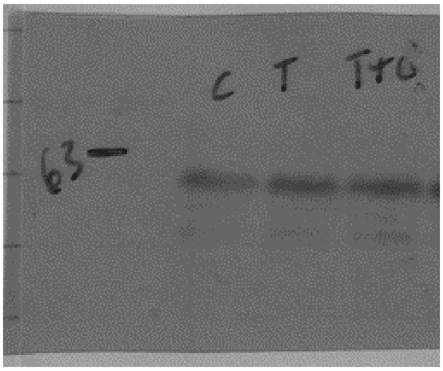

Total Smad3

K

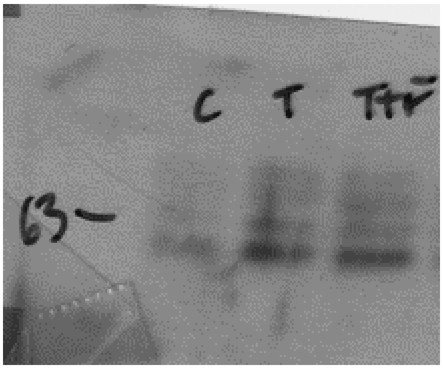

pSmad3

63 -

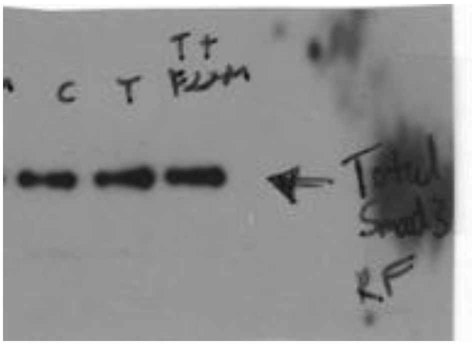

Figure 4

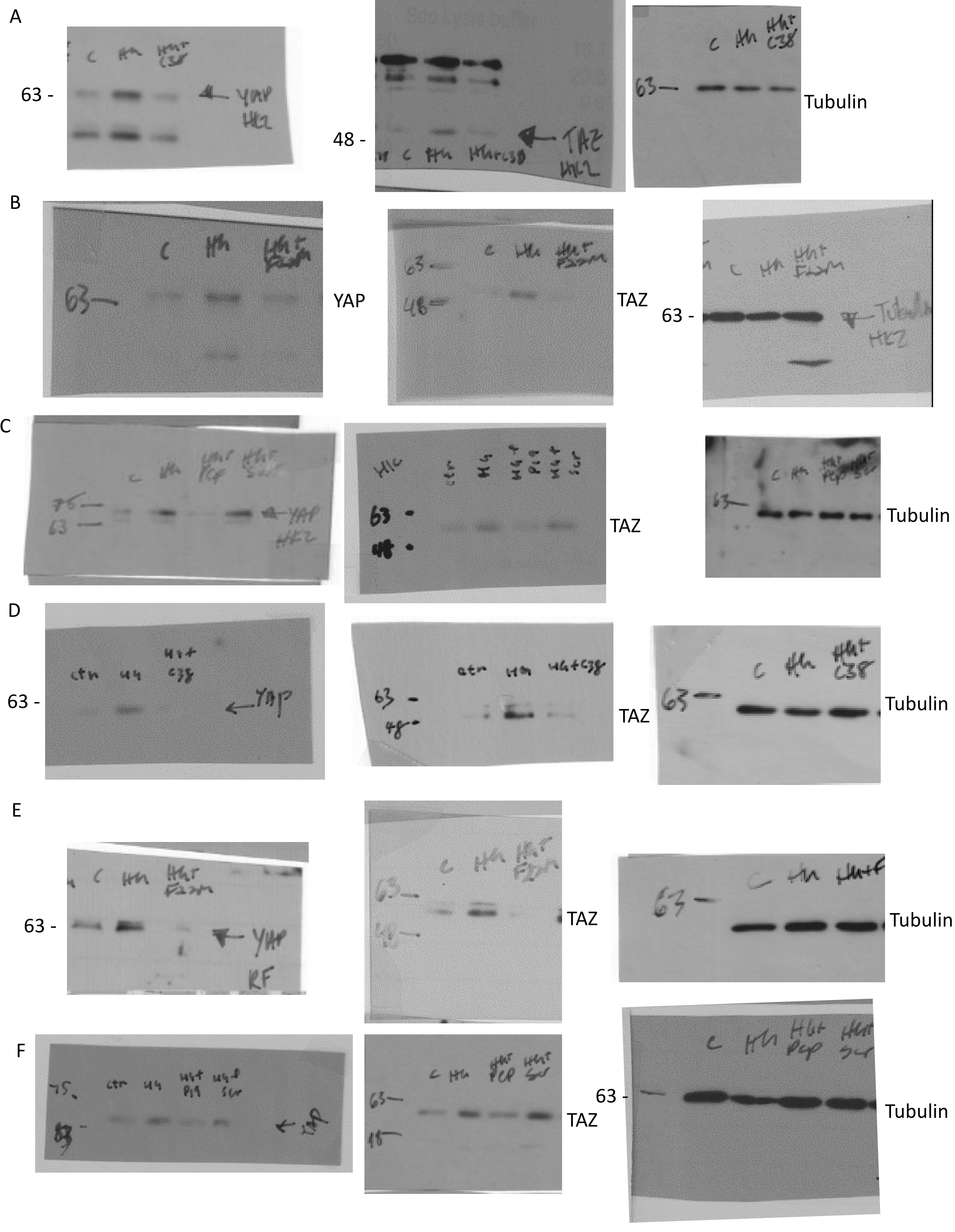
A
63 -
48 -
Tubulin
B
YAP
TAZ
63 -
C
TAZ
Tubulin
D
63 -
TAZ
Tubulin
E
63 -
TAZ
Tubulin
F
TAZ
63 -
Tubulin

Figure 4

G

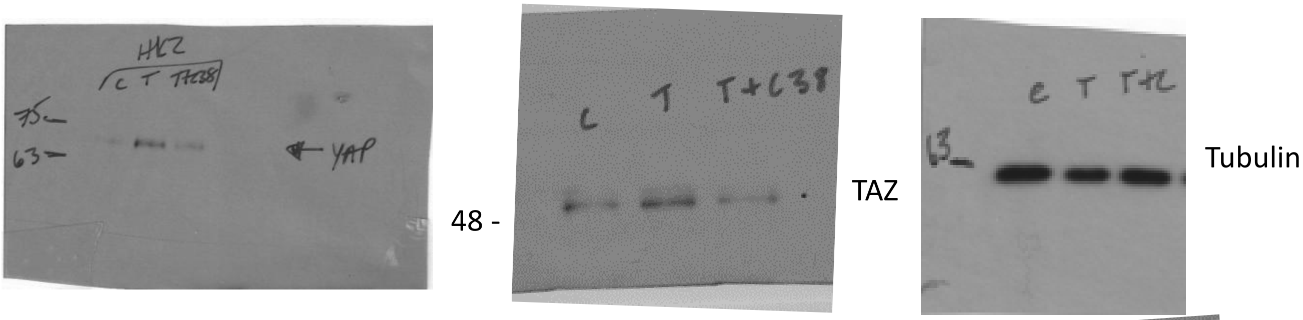

H

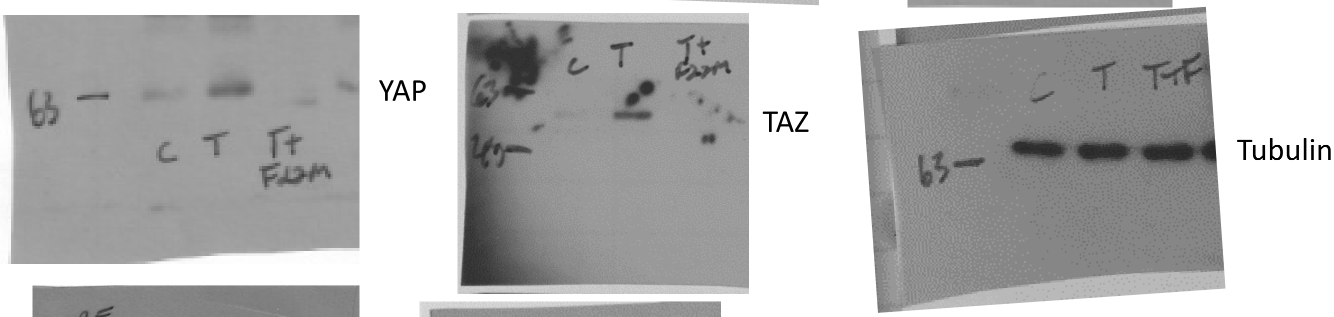

J

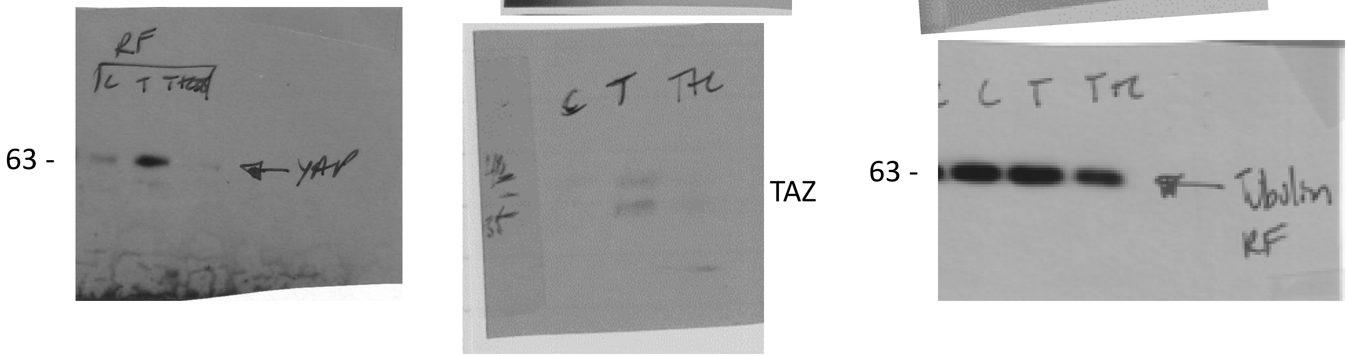

K

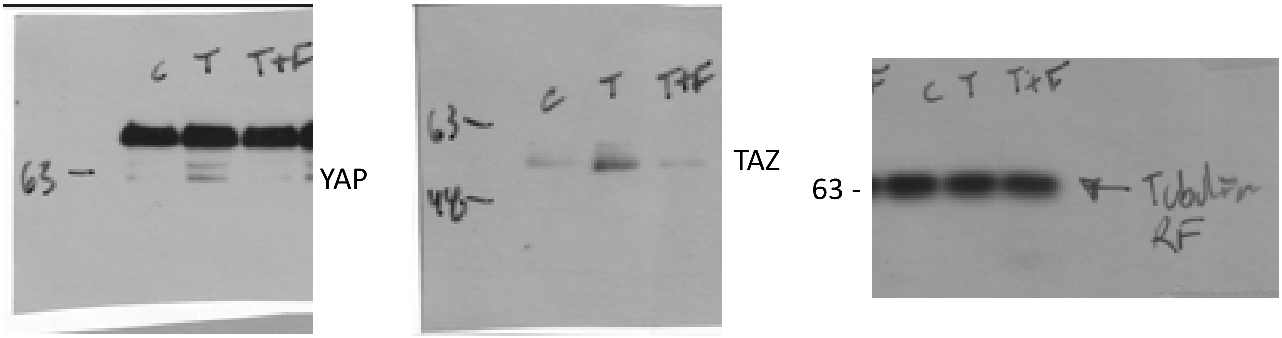

Figure 5

A

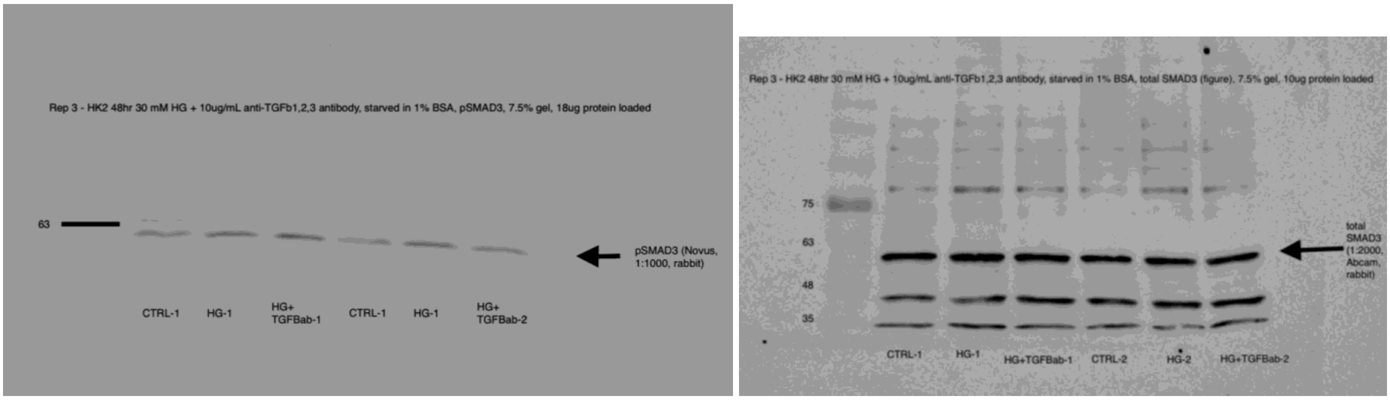

Rep 3 - HK2 48hr 30 mM HG + 10ug/mL anti-TGFb1,2,3 antibody, starved in 1% BSA, pSMAD3, 7.5% gel, 18ug protein loaded
63
pSMAD3 (Novus, 1:1000, rabbit)
CTRL-1
HG-1
HG+ TGFBab-1
CTRL-1
HG-1
HG+ TGFBab-2
Rep 3 - HK2 48hr 30 mM HG + 10ug/mL anti-TGFb1,2,3 antibody, starved in 1% BSA, total SMAD3 (figure), 7.5% gel, 10ug protein loaded
75
63
48
35
total SMAD3 (1:2000, Abcam, rabbit)
CTRL-1
HG-1
HG+TGFBab-1
CTRL-2
HG-2
HG+TGFBab-2

B

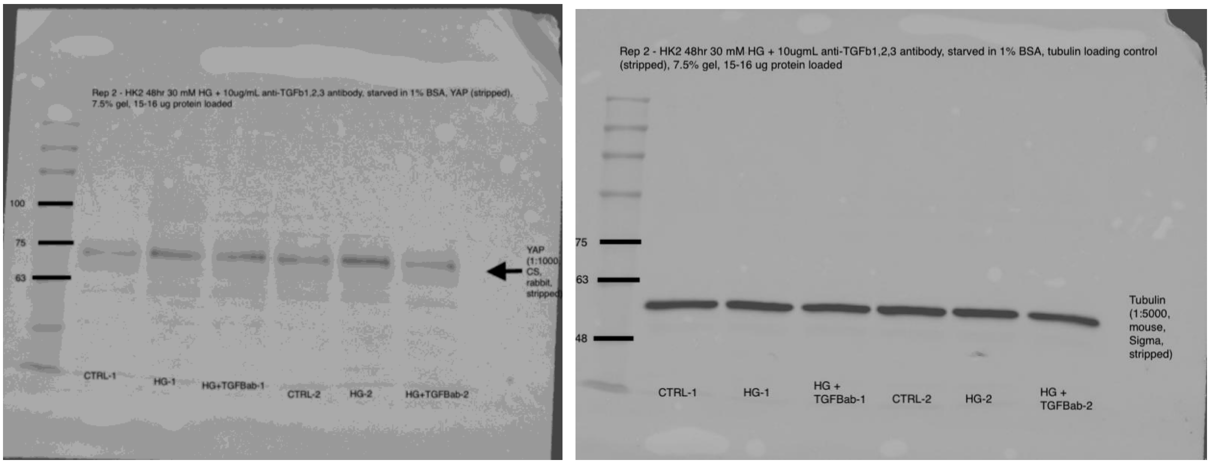

Rep 2 - HK2 48hr 30 mM HG + 10ug/mL anti-TGFb1,2,3 antibody, starved in 1% BSA, YAP (stripped), 7.5% gel, 15-16 ug protein loaded
100
75
63
YAP (1:1000 CS, rabbit, stripped)
CTRL-1
HG-1
HG+TGFBab-1
CTRL-2
HG-2
HG+TGFBab-2
Rep 2 - HK2 48hr 30 mM HG + 10ugmL anti-TGFb1,2,3 antibody, starved in 1% BSA, tubulin loading control (stripped), 7.5% gel, 15-16 ug protein loaded
75
63
48
Tubulin (1:5000, mouse, Sigma, stripped)
CTRL-1
HG-1
HG + TGFBab-1
CTRL-2
HG-2
HG + TGFBab-2

Supplementary Figure 1

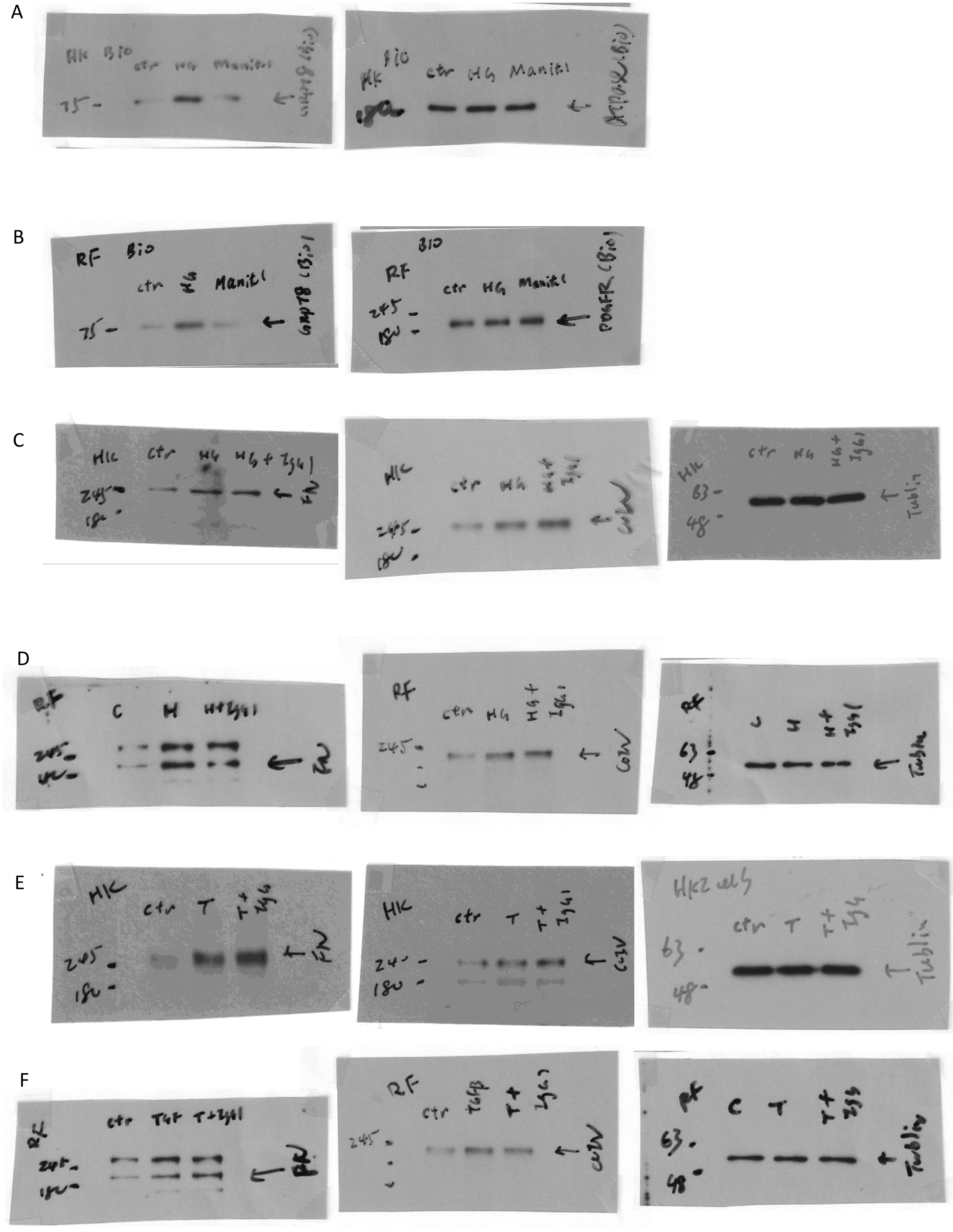

Supplementary Figure 4

A

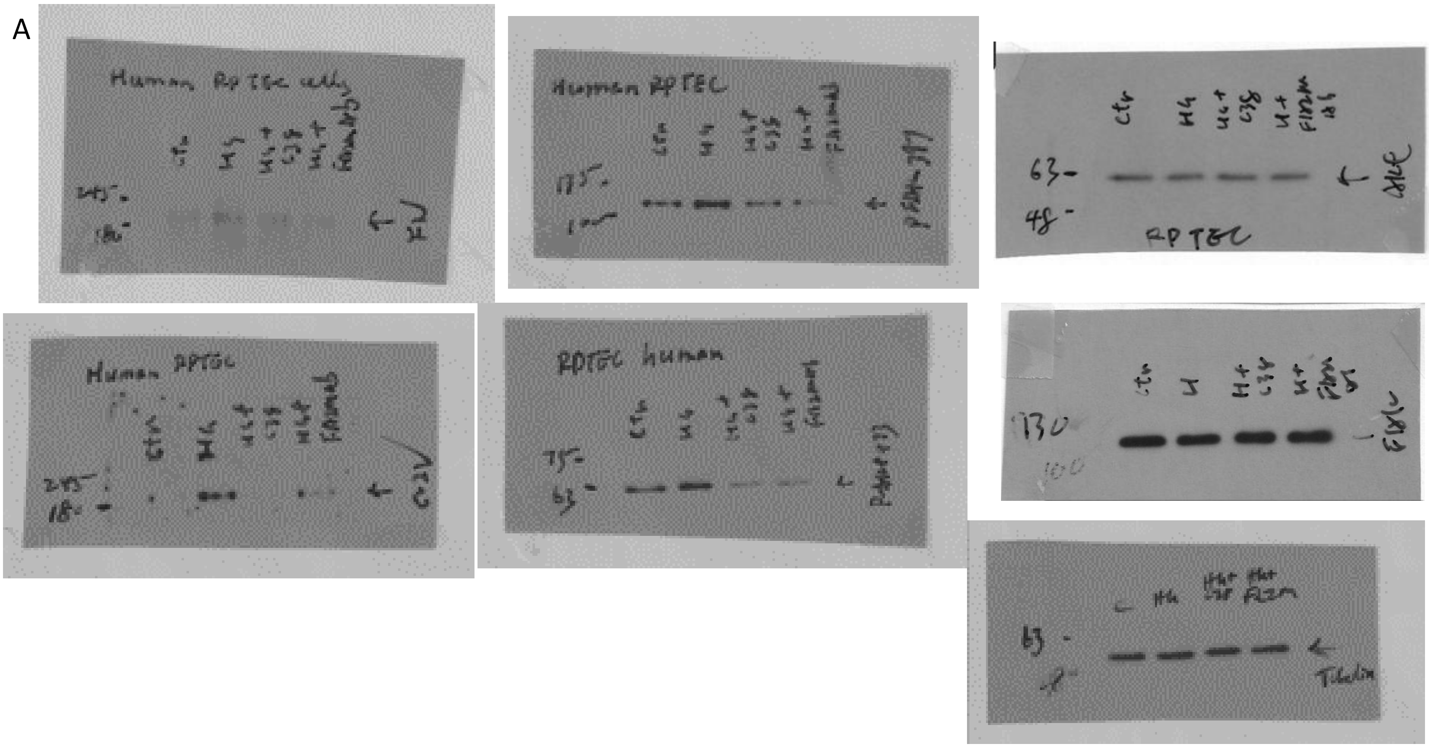

B

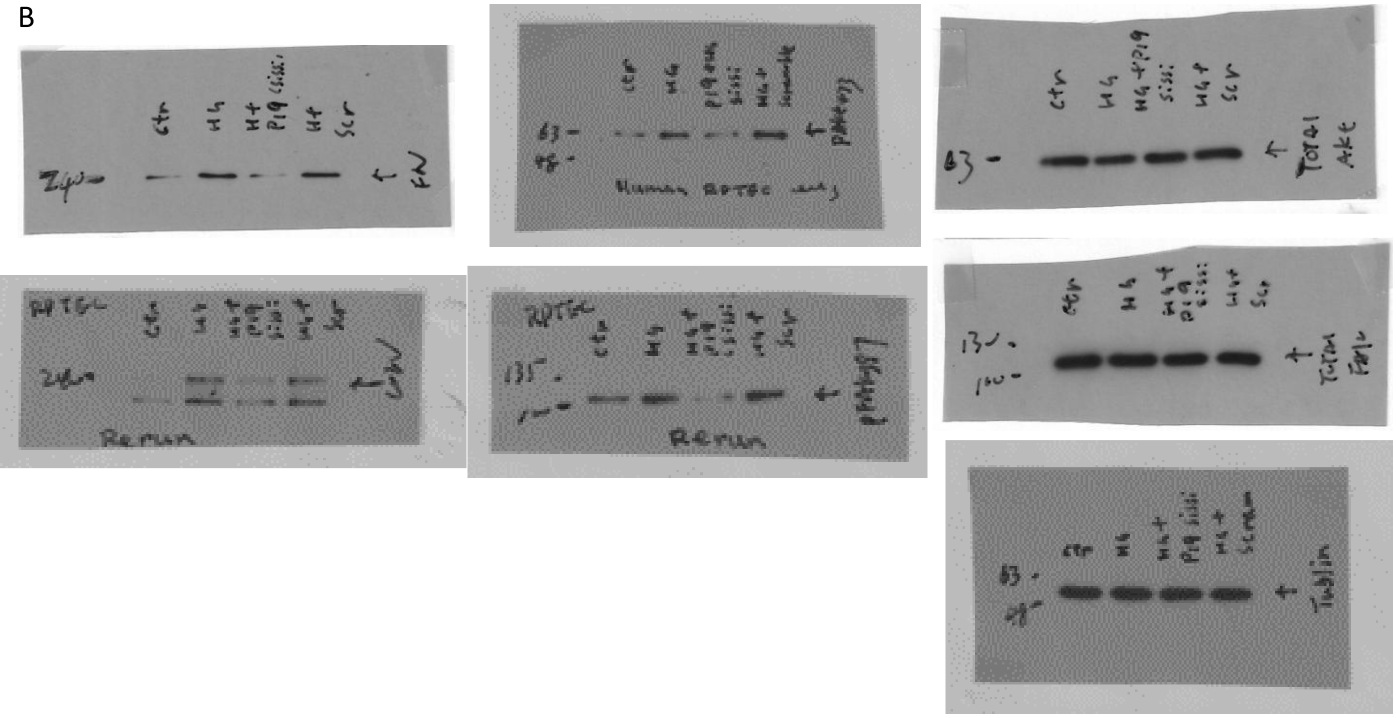

Supplement: Unedited blot and gel images [file jciinsight-10-183998-s054.pdf]
